# The EAT-Lancet Commission’s Planetary Health Diet Compared With the Institute for Health Metrics and Evaluation Global Burden of Disease Ecological Data Analysis

**DOI:** 10.7759/cureus.40061

**Published:** 2023-06-06

**Authors:** David K Cundiff, Chunyi Wu

**Affiliations:** 1 Global Burden of Disease, Institute for Health Metrics and Evaluation, Long Beach, USA; 2 Statistics, Michigan Medicine, Ann Arbor, USA

**Keywords:** ai & robotics in healthcare, population-attributable risk percents, global burden of disease (gbd), eat-lancet planetary health diet, noncommunicable diseases, multivariable regression, diet quality, dietary recommendations

## Abstract

Background

This article aimed to compare the EAT-Lancet Commission’s “Planetary Health Diet” (PHD) with the Institute for Health Metrics and Evaluation (IHME) Global Burden of Disease Study 1990-2017 (GBD2017) dietary and other risk factor data. In the PHD/GBD comparison, we also intended to show the relevance of a new multiple regression analysis methodology with dietary and non-dietary risk factors (independent variables) for noncommunicable disease (NCD) deaths/100000/year in males and females 15-69 years old from 1990 to 2017 (NCDs, dependent variable).

Methods

We formatted worldwide GBD2017 dietary risk factors and NCD data on 1120 worldwide cohorts to obtain 7846 population-weighted cohorts. Each cohort represented about one million people, totaling about 7.8 billion people from 195 countries. With an empirically derived methodology, we compared the PHD animal- and plant-sourced food recommended ranges (kilocalories/day=KC/d) with optimal dietary ranges (KC/d) from GBD cohort data. Using GBD data subsets with low and high animal food consumption cohorts, our new GBD multiple regression formula derivation methodology equated risk factor formula coefficients to their population-attributable risk percents (PAR%s).

Results

We contrasted PHD recommendations for the available 14 dietary risk factors (KC/d means and ranges) with our GBD analysis methodology’s optimal ranges for each dietary variable (KC/d mean and range): PHD beef, lamb, and pork mean: 30 KC/d (range: 0-60 KC/d)/GBD processed meat: 8.86 (1.69-16.03)+GBD red meat: 44.52 (20.37-68.68), PHD fish: 40 (0-143)/GBD: 19.68 (3.45-35.90), PHD whole milk or equivalents: 153 (0-306)/GBD: 40.00 (18.89-61.11), PHD poultry: 62 (0-124)/GBD: 56.10 (24.13-88.07), PHD eggs: 19 (0-37)/GBD: 19.42 (9.99-28.86), PHD: saturated oils 96 (0-96)/GBD added saturated fatty acids (SFA): 116.55 (104.04-129.07), PHD all added sugars: 120 (0-120)/GBD sugary beverages: 286.37 (256.99-315.76), PHD tubers or starchy vegetables: 39 (0-78)/GBD potatoes: 84.16 (75.75-92.58)+GBD sweet potatoes: 9.21 (4.05-14.37), PHD fruits: 126 (63-189)/GBD: 63.03 (21.61-113.71), PHD vegetables: 78.32 (9.48-196.14)/GBD: 85.05 (66.75-103.36), PHD nuts: 291 (0-437)/GBD nuts and seeds: 10.97 (5.95-15.98), PHD whole grains: 811 (811/811)/GBD: 56.14 (50.53-61.76), PHD legumes: 284 (0-379)/GBD: 59.93 (45.43-74.43), and total animal food PHD: (0/400)/GBD: 329.84 (212.49-447.19). Multiple regression low and high animal food subsets’ (animal foods mean=147.09 KC/d versus animal foods mean=482.00 KC/d) formulas each with 28 dietary and non-dietary risk factors (independent variables) accounted for 52.53% and 28.83% of their respective total formula PAR%s with NCDs (dependent variable).

Conclusions

GBD data modeling supported many but not all the PHD dietary recommendations. GBD data suggested that the amount of consumption of animal foods was the dominant determinate of NCDs of countries globally. Adding to the univariate associations, multiple regression risk factor formulas with risk factor coefficients equated to their PAR%s further elucidated dietary influences on NCDs. This paper and the soon-to-be-released IHME GBD2021 (1990-2021) data should help inform the EAT-Lancet 2.0 Commission’s work.

## Introduction

The “Planetary Health Diet” (PHD), proposed in 2019 by the EAT-Lancet Commission [1], was meant to spark a global transformation in human diets to improve health and mitigate climate change. The article “Food in the Anthropocene: the EAT-Lancet Commission on healthy diets from sustainable food systems” [2] detailed a range of animal- and plant-based foods for which they derived optimal dietary ranges (kilocalories/day or KC/d) to benefit human health and environmental sustainability. The PHD was the first and only serious attempt to inform policymakers, influencers, and the public about food consumption beneficial for global human health and ecological sustainability.

The report said, “Transformation to healthy diets by 2050 will require substantial dietary shifts, including a greater than 50% reduction in global consumption of unhealthy foods such as red meat and sugar, and a greater than 100% increase in the consumption of healthy foods such as nuts, fruits, vegetables and legumes” [1].

This paper will compare the PHD recommendations for healthy diets by 2050 with findings from statistical analyses of the Global Burden of Disease Study 1990-2017 (GBD2017) data from the Institute for Health Metrics and Evaluation (IHME).

For the scientific target to measure the effects of healthy versus unhealthy quantities of animal- and plant-sourced foods (KC/d), we chose the rate of noncommunicable disease (NCD) deaths/100000/year in males and females 15-69 years old. According to the World Health Organization (WHO) [3], noncommunicable diseases kill about 41 million people per year (74% of all deaths), of which 17.9 million deaths occur before the age of 70 (32% of all deaths).

Research in context

Evidence Before This Study

Regarding the optimal diet for overall human health, there is no consensus among nutrition professionals, people from different countries or cultures, or people within the same family. The EAT-Lancet Commission’s methodology of validating their Planetary Health Diet (PHD) was an expert opinion. The EAT-Lancet Commission cited no previous examples of published efforts to define ranges of consumption of foods in an optimal human diet, mentioning the lack of scientific targets to define an overall healthy diet. The PHD was the first and only serious attempt to inform policymakers, influencers, and the public about food consumption beneficial for global human health and ecological sustainability.

Added Value of This Study

This study used GBD worldwide data including 20 dietary risk factors (KC/d) and two combination dietary risk factors correlated with rates/100000/year of early deaths (ages 15-69 years) from over 100 noncommunicable diseases (NCDs, e.g., diseases of the heart, lung, liver, kidney, and brain and cancers). Since NCDs account for 39% of all human deaths, rates of NCDs qualify as scientific targets to test ranges of consumption of foods. With low animal food and high animal food consumption (KC/d) subsets of global cohorts with their respective NCDs, we devised statistical methodologies to derive optimal dietary range estimates. The methodologies included generating multiple regression risk factor formulas with >20 risk factor coefficients equated to their respective population-attributable risk percents (PAR%s). We compared optimal KC/d range estimates in the PHD with GBD data-derived estimates. Our methodologies addressed ecological fallacy, multicollinearity, and other confounding.

Implications of All the Available Evidence

GBD data supported many but not all the food optimal range (KC/d) estimates of the PHD. Twenty out of 44 countries within the lowest NCD group had mean animal foods of <400 KC/d, which fits with EAT-Lancet Commission’s primary contention. Those countries had far fewer early cancer deaths than countries with mean animal foods of ≥400 KC/d. Our methodology-derived multiple regression risk factor formulas had risk factor coefficients equated to their PAR%s. This can be used with any noncommunicable disease in the GBD database. This paper and the soon-to-be-released GBD2021 (1990-2021) data analysis should help inform the EAT-Lancet 2.0 Commission’s work.

## Materials and methods

As volunteer collaborators with the IHME, we acquired and utilized raw GBD worldwide ecological data (n=1120 male and female cohorts, from 195 countries and 365 subnational locations). Data included rates of NCDs, dietary and non-dietary risk factors, and covariates of male and female cohorts 15-49 years old and 50-69 years old from each of the 28 years (1990-2017). GBD2021 risk factor and health outcome ecological data inputs including worldwide citations of over 12000 surveys (updated from GBD2017 and GBD2019) will soon be available online from the IHME [4].

The main characteristics of the IHME GBD data sources, the protocol for the GBD study, and all risk factor values have been published by IHME GBD data researchers and discussed elsewhere [5]. These include detailed descriptions of categories of input data, potentially important biases, and methodologies of analysis. We did not clean or preprocess any of the GBD data. GBD cohort risk factor and health outcome data from the IHME had no missing records other than dietary covariates (poultry, eggs, potatoes, corn, rice, and sweet potatoes) for the United States.

Table 1 lists the relevant GBD dietary risk factors and covariates with definitions of those risk factor exposures [6].

**Table 1:** Definitions of GBD risk factors and covariates related to NCDs SD, standard deviation; WHO, World Health Organization; ACR, albumin-to-creatinine ratio; GFR, glomerular filtration rate; MET, metabolic equivalent of task; GBD, Global Burden of Disease; NCD, noncommunicable disease; KC/d, kilocalories/day

Variables	Definition
Alcohol	Any alcohol consumption (KC/d)
Ambient particulate matter pollution	Annual average daily exposure to outdoor air concentrations of particulate matter with an aerodynamic diameter of ≤2.5 μg/m^3^ (PM_2.5_)
Body mass index	Body mass index (BMI) (kg/m^2^): the dependent variable of interest
Child underweight	The proportion of children -3 SD to -2 SD of the WHO 2006 standard weight-for-age curve (0-1)
Corn	Corn availability per capita (KC/d), a covariate
Discontinued breast feeding	The proportion of children aged 6-23 months who do not receive any breast milk
Eggs	Egg availability per capita (KC/d), a covariate
Fasting plasma glucose	Fasting plasma glucose (mmol/L)
Fish	This variable expressed in KC/d was derived by determining the weight of fish in gram corresponding to 1 g of omega-3 fatty acids (eicosapentaenoic acid and docosahexaenoic acid) by averaging the fish grams per 1 g of omega-3 fatty acids in 20 species of fish=117.04 KC/d fish/1 KC/d omega-3 fatty acids (Table 2)
Fruits	The consumption of fruits (includes fresh, frozen, cooked, canned, or dried fruit, but excludes fruit juices and salted or pickled fruits) (KC/d)
Household air pollution from solid fuels	Individual exposure to PM_2.5_ due to the use of solid cooking fuel
Kidney function impaired	Proportion of the population with ACR of >30 mg/g or GFR of <60 mL/minute/1.73 m^2^ and stage III kidney failure, excluding end-stage renal disease
Kilocalories available/day	The mean number of kilocalories per capita available per day to people in each location (KC/d available), a covariate
Low-density lipoprotein (LDL) cholesterol	Serum low-density lipoprotein cholesterol (mmol/L)
Lead exposure	Blood lead levels in μg/dL of blood; bone lead levels in μg/g of the bone
Legumes	The consumption of beans, lentils, and pulses (KC/d)
Milk	The consumption of milk including nonfat, low-fat, and full-fat milk but excluding soy milk and other plant derivatives (KC/d)
Nuts and seeds	The consumption of nuts and seeds (KC/d)
Physical activity	Average weekly physical activity at work and home and transport-related and recreational measured by MET per minute per week. Less than 3000 METs per week constitutes low physical activity
Poultry	Poultry availability per capita (KC/d), a covariate
Potatoes	Potato availability per capita (KC/d), a covariate
Processed meat	The consumption of any processed meat (includes meat preserved by smoking, curing, salting, or the addition of chemical preservatives, including bacon, salami, sausages, or deli or luncheon meats such as ham, turkey, and pastrami) (KC/d)
Red meat	The consumption of red meat (includes beef, pork, lamb, and goat, but excludes poultry, fish, eggs, and all processed meats) (KC/d)
Rice	Rice availability per capita (KC/d), a covariate
Seafood omega-3 fatty acids	Seafood omega-3 fatty acids (eicosapentaenoic acid and docosahexaenoic acid) in tablet or fish form (gram/day, convertible to fish KC/d)
Secondhand smoke	Average daily exposure to air particulate matter from secondhand smoke with an aerodynamic diameter smaller than 2.5 μg, measured in μg/m^3^, among nonsmokers
Smoking	The prevalence of the current use of any smoked tobacco product
Sociodemographic index (SDI)	SDI is a composite indicator of development status that was originally constructed for GBD2015 and is derived from components that correlate strongly with health outcomes. It is the geometric mean for indices of the total fertility rate among females younger than 25 years, mean education for those aged 15 years or older, and lag-distributed income per capita. The resulting metric ranges from 0 to 1, with higher values corresponding to higher levels of development
Sublingual tobacco	The current use of any chewing tobacco product
Sugar-sweetened beverages	The consumption of any beverage with ≥50 calories of sugar per one cup serving, including carbonated beverages, sodas, energy drinks, and fruit drinks but excluding 100% fruit and vegetable juices (KC/d)
Sweet potatoes	Sweet potato availability per capita (KC/d), a covariate
Systolic blood pressure	Systolic blood pressure (mm Hg)
Vegetables	The consumption of frozen, cooked, canned, or dried vegetables (including legumes but excluding salted or pickled, juices, nuts and seeds, and starchy vegetables such as potatoes or corn) (KC/d)
Vitamin A deficiency	The proportion of children aged 0-5 years with serum retinol concentration of <0.7 μmol/L
Whole grains	The consumption of whole grains (bran, germ, and endosperm in their natural proportions) from breakfast cereals, bread, rice, pasta, biscuits, muffins, tortillas, pancakes, and others (KC/d)

Food risk factors came from surveys that IHME researchers utilized as grams/day (g/day) consumed on average. GBD dietary covariate data originally came from Food and Agriculture Organization [7] surveys of animal and plant food commodities available per capita in countries worldwide (i.e., potatoes, corn, rice, sweet potatoes, poultry, and eggs), as opposed to consumed on average.

Study design and population

For NCDs, with dietary risk factors, non-dietary risk factors, and combinations of dietary or non-dietary risk factors, we averaged the values for ages 15-49 years old together with 50-69 years old for each male and female cohort for each year. Finally, for each male and female cohort, we averaged data from all 28 years (1990-2017) of the means of the rate of NCDs and dietary and other risk factor exposures using the computer software program R (R Foundation for Statistical Computing, Vienna, Austria).

World population data from the World Bank or the Organization for Economic Co-operation and Development could not be used because they did not include all 195 countries or any subnational data. To weigh the data according to population, internet searches (mostly Wikipedia) yielded the most recent population estimates for countries and subnational states, provinces, and regions. The 1120 GBD cohorts available were population-weighted by the software program R, resulting in an analysis dataset with 7846 population-weighted cohorts, representing about 7.8 billion people projected for 2019. Each male or female cohort in the population-weighted analysis dataset represented approximately one million people (range: from <100000 to 1.5 million).

Table 2 details how omega-3 fatty acid gram/day was converted to fish gram/day using data on the omega-3 fatty acid content of frequently eaten fish from the National Institutes of Health Office of Dietary Supplements (the United States) [8].

**Table 2:** Omega-3 fatty acid gram to fish gram calculation ^a^Data on omega-3 fatty acid content of varieties of fish came from the National Institutes of Health Office of Dietary Supplements (the United States) DHA, docosahexaenoic acid; EPA, eicosapentaenoic acid

Fish^a^	DHA (gram/3 oz fish)	EPA (gram/3 oz fish)	Omega-3 fatty acid (FA) (DHA and EPA) (gram/3 oz fish) mean	Fish (3 oz=85.02 g)	Fish (gram) per omega-3 fatty acids (gram)=columns E/F
Salmon: Atlantic, farmed	1.24	0.59			
Salmon: Atlantic, wild	1.22	0.35			
Herring: Atlantic	0.94	0.77			
Sardines: canned in tomato sauce, drained	0.74	0.45			
Mackerel: Atlantic	0.59	0.43			
Salmon: pink, canned, drained	0.63	0.28			
Trout: rainbow, wild	0.44	0.40			
Oysters: eastern, wild	0.23	0.30			
Sea bass	0.47	0.18			
Shrimp	0.12	0.12			
Lobster	0.07	0.10			
Tuna: light, canned in water, drained	0.17	0.02			
Tilapia	0.11				
Scallops	0.09	0.06			
Cod: Pacific	0.1	0.04			
Tuna: yellowfin	0.09	0.01			
Mean DHA and EPA, omega-3 fatty acid gram/3 oz fish	0.4531	0.2733			
Calculations of total omega-3 FA gram to fish gram			0.726	85.02	117.04

As shown in Table 3, we converted all of the animal and plant food data, including alcohol and sugary beverage consumption, from gram/day to KC/d. For the gram/day to KC/d conversions, we used the Nutritionix Track app (Nutritionix LLC, Washington, DC) [9], which tracks types and quantities of foods consumed.

**Table 3:** Calculations of kilocalories/day (KC/d) from grams/day (g/day) of animal and plant foods ^b^Source: Nutritionix application

Foods^b^	Food subcategories	KC/serving	Gram/serving	KC/gram
Milk (2% fat)		122	244	0.50
Fish		218	170	1.28
Eggs		72	50	1.44
Poultry		187	85	2.91
Red meat		247	85	2.91
Processed meat				
	Salami	222	59	3.76
	Pastrami	104	71	1.46
	Ring baloney	86	28	3.07
	Pepperoni	94	100	0.94
Average processed meat		126.5	64.5	1.96
Fruits		97	162	0.60
Vegetables		59	91	0.65
Legumes		249	179	1.39
Nuts		172	28	6.14
Seeds				
	Flax seeds	55	10	5.50
	Chia seeds	58	12	4.83
	Fennel seeds	34.5	10	3.45
	Hemp seeds	55.3	10	5.53
Average of seeds		50.7	10.5	4.83
Average of nuts and seeds		111.4	19.25	5.78
Corn		99	103	0.96
Potatoes		161	173	0.93
Sweet potatoes		115	151	0.76
Rice		205	158	1.30
Whole grains		120	52	2.31

Saturated fatty acids (SFA: 0-1 portion of the entire diet KC/d) was not available with GBD2017 data, so we used GBD SFA risk factor data from GBD2016. Polyunsaturated fatty acid (PUFA) and trans fatty acid (TFA) GBD risk factor data from 2017 (0-1 portion of the entire diet KC/d) were also utilized, but monounsaturated fat data were not available. These fatty acid data expressed for each cohort as 0-1 portion of the entire diet were converted to KC/d by multiplying by the total KC/d available per capita (a covariate from the Food and Agriculture Organization [7]).

Outcome variable, dietary and other risk factors, and covariates

NCDs, the principal outcome variable of this analysis, were a combination variable consisting of the deaths/100000/year of male and female cohorts 15-69 years old from (1) cardiovascular diseases, (2) type 1 diabetes, (3) type 2 diabetes, (4) chronic respiratory disease, (5) chronic renal disease, (6) liver cirrhosis, (7) inflammatory bowel disease, (8) liver cancer, (9) esophageal cancer, (10) stomach cancer, (11) prostate cancer, (12) breast cancer, (13) bladder cancer, (14) non-Hodgkin’s lymphoma, (15) ovarian cancer, (16) brain cancer, (17) lung cancer, (18) multiple myelomas, (19) colorectal cancer, (20) kidney cancer, (21) melanoma, (22) pancreatic cancer, and (23) many other less common noncommunicable diseases.

Dietary and other risk factors included (1) processed meat (KC/d), (2) red meat (KC/d), (3) fish (KC/d), (4) milk (including all dairy products) (KC/d), (5) poultry (KC/d) available (covariate), (6) eggs (KC/d) available (covariate), (7) added saturated fatty acids (added SFA) (KC/d), (8) added polyunsaturated fatty acids (added PUFA) (KC/d), (9) added trans fatty acids (added TFA) (KC/d), (10) alcohol (KC/d), (11) sugary beverages (KC/d), (12) potatoes (KC/d) available (covariate), (13) sweet potatoes (KC/d) available (covariate), (14) corn (KC/d) available (covariate), (15) fruits (KC/d), (16) vegetables (KC/d), (17) nuts and seeds (KC/d), (18) whole grains (KC/d), (19) legumes (KC/d), (20) rice (KC/d) available (covariate), (21) animal food seven (KC/d) (processed meat {KC/d}+red meat {KC/d}+fish {KC/d}+milk {KC/d}+poultry {KC/d}+eggs {KC/d}+added SFA {KC/d}), (22) healthy plant seven (KC/d) (fruits {KC/d}+vegetables {KC/d}+nuts and seeds {KC/d}+whole grains {KC/d}+legumes {KC/d}+sweet potatoes {KC/d}+added PUFA {KC/d}), (23) total KC/d available (covariate), (24) vitamin A deficiency in children <5 years old/100000/year, (25) sodium (gram/day), (26) calcium (gram/day), (27) dietary fiber (gram/day), (28) physical activity metabolic equivalent of tasks (METs), (29) child underweight of >2 standard deviation (SD) below mean for age, (30) stop breast feeding in <6 months, (31) ambient air pollution (PM_0.25_), (32) smoking rate (0-1), (33) secondhand smoking (0-1), (34) sublingual tobacco rate (0-1), (35) blood lead level (mcg/dL), (36) household air pollution (0-1), (37) kidney disease stage III (0-1), (38) body mass index (BMI) (kg/m^2^), (39) low-density lipoprotein cholesterol (LDLc) (mmol/L), (40) fasting plasma glucose (FPG) (mmol/L), (41) systolic blood pressure (SBP) (mm Hg), (42) sociodemographic index (SDI) (0-1), and (43) sex (male=1, and female=2).

As noted above, we created two combination variables: (1) “Animal food seven (KC/d)”=processed meats (KC/d)+red meats (KC/d)+fish (KC/d)+milk-derived foods (KC/d)+poultry (KC/d)+eggs (KC/d)+added SFA (KC/d). In accordance with the Harvard School of Public Health [10], we considered that animal foods were higher in SFA than plant-based foods, so added SFA was included in the animal food seven combination risk factor. Creating animal food seven will make a comparison with the animal-sourced food variable in the PHD possible. (2) “Healthy plant seven (KC/d)”=fruits (KC/d)+vegetables (KC/d)+nuts and seeds (KC/d)+whole grains (KC/d)+legumes (KC/d)+sweet potatoes (KC/d)+added PUFA (KC/d). Plant foods are higher in PUFA than animal foods, so we included added PUFA with healthy plant foods.

We did not include three of the plant food covariates (potatoes, corn, and rice) with the healthy plant foods because of the following: (1) Half or more of potatoes available worldwide were ultra-processed and contained many additives [11]. (2) Corn available included high-fructose corn syrup as demonstrated by the high correlation of corn with sugary beverages in this database (r=0.330, 95% confidence interval {CI}=0.310-0.349, p<0.0001). (3) Rice available was mostly refined without bran (the fibrous outer layer) and germ (the nutritious core). Whole grain rice was included in the analysis with the whole grains.

The animal foods and plant foods and beverages mostly contained some SFA, PUFA, and TFA. The GBD variables did not distinguish the added fatty acids in oils and fats (e.g., vegetable oil, butter, and lard) from fatty acids that were in whole foods (e.g., red meat, fruits, and sweet potatoes). In order not to double count the KC/d of SFA, PUFA, and TFA in animal and plant foods versus added oils and fats, we used dietary composition data from the website Our World in Data [12]. By using data from “macronutrient composition” and from “food composition,” we calculated the approximate amount of KC/d of added fatty acids. To do this, we downloaded the daily average availability of total fat (KC/d) and the availability of added fats and oils (KC/d) for the 169 countries included in the GBD data. We then divided the added fats and oils by the total fat to get the ratio of added fats and oils available to total available fat. From that ratio and the total SFA, PUFA, and TFA, we derived the variables added SFA, added PUFA, and added TFA. Table 4 shows the countries with data, including oils and fats, total fat, and the ratio of oils and fats to total fat.

**Table 4:** Calculations for the proportions of total fat from added fats and oils ^c^From Our World in Data FAO, Food and Agriculture Organization; KC/d, kilocalories/day

Countries^c^	Oil and fat (FAO {2017}, KC/d available per person per day) mean values in 1990-2013	Total KC/d available from fat (FAO {2017}, mean values in 1990-2013)	Oils and fats KC/d/total fat KC/d
Afghanistan	134	311	0.431
Albania	280	760	0.368
Algeria	386	610	0.633
Angola	239	389	0.614
Antigua and Barbuda	301	743	0.405
Argentina	420	994	0.423
Armenia	373	870	0.429
Australia	660	1217	0.542
Austria	908	1439	0.631
Azerbaijan	153	406	0.377
Bahamas	304	814	0.373
Bangladesh	148	229	0.646
Barbados	436	849	0.514
Belarus	444	965	0.460
Belgium	1005	1467	0.685
Belize	349	632	0.552
Benin	299	404	0.740
Bermuda	388	1023	0.379
Bolivia	187	408	0.458
Bosnia and Herzegovina	188	547	0.344
Botswana	264	493	0.535
Brazil	502	878	0.572
Brunei	333	700	0.476
Bulgaria	470	847	0.555
Burkina Faso	316	484	0.653
Cabo Verde	334	644	0.519
Cambodia	120	277	0.433
Cameroon	318	424	0.750
Canada	859	1280	0.671
Central African Republic	422	567	0.744
Chile	293	725	0.404
China	284	689	0.412
Colombia	356	640	0.556
Congo	346	440	0.786
Costa Rica	437	724	0.604
Cote d’Ivoire	382	474	0.806
Croatia	439	825	0.532
Cuba	263	517	0.509
Cyprus	494	1003	0.4925
Czechia	657	1103	0.596
Denmark	652	1197	0.545
Djibouti	268	433	0.619
Dominica	304	721	0.422
Dominican Republic	459	686	0.669
Ecuador	444	752	0.596
Egypt	249	523	0.476
El Salvador	242	508	0.476
Estonia	302	827	0.365
Eswatini	188	403	0.467
Ethiopia	68	185	0.368
Fiji	616	870	0.708
Finland	426	1162	0.367
France	757	1481	0.511
French Polynesia	556	1033	0.538
Gabon	299	485	0.616
Gambia	520	641	0.811
Georgia	195	488	0.400
Germany	779	1274	0.611
Ghana	275	371	0.741
Greece	790	1322	0.598
Grenada	404	762	0.530
Guatemala	207	441	0.469
Guinea	381	487	0.782
Guinea-Bissau	376	513	0.733
Guyana	261	471	0.554
Haiti	220	341	0.645
Honduras	312	599	0.521
Hungary	716	1192	0.601
Iceland	396	1205	0.329
India	282	417	0.676
Indonesia	325	432	0.752
Iran	376	619	0.607
Iraq	372	522	0.713
Ireland	581	1190	0.488
Israel	768	1200	0.640
Italy	846	1374	0.616
Jamaica	398	704	0.565
Japan	521	788	0.661
Jordan	496	775	0.640
Kazakhstan	368	850	0.433
Kenya	193	428	0.451
Kiribati	773	896	0.863
Kuwait	484	952	0.508
Kyrgyzstan	141	525	0.269
Laos	86	261	0.330
Latvia	514	966	0.532
Lebanon	643	963	0.668
Lesotho	61	305	0.200
Liberia	436	513	0.850
Lithuania	342	788	0.434
Luxembourg	420	1318	0.319
Madagascar	92	252	0.365
Malawi	122	282	0.433
Malaysia	466	777	0.600
Maldives	271	511	0.530
Mali	245	510	0.480
Malta	453	979	0.463
Mauritania	327	619	0.528
Mauritius	505	764	0.661
Mexico	351	774	0.453
Moldova	258	559	0.462
Mongolia	180	721	0.250
Montenegro	231	709	0.326
Morocco	353	555	0.636
Mozambique	223	343	0.650
Myanmar	268	431	0.622
Namibia	164	401	0.409
Nepal	206	373	0.552
Netherlands	624	1240	0.503
New Caledonia	552	1014	0.544
New Zealand	585	1075	0.544
Nicaragua	258	457	0.565
Niger	213	421	0.506
Nigeria	427	541	0.789
North Korea	201	326	0.617
North Macedonia	491	805	0.610
Norway	658	1282	0.513
Oman	337	673	0.501
Pakistan	366	603	0.607
Panama	356	604	0.589
Paraguay	410	774	0.530
Peru	218	401	0.544
Philippines	192	424	0.453
Poland	538	1024	0.525
Portugal	693	1190	0.582
Romania	387	863	0.448
Russia	375	783	0.479
Rwanda	97	192	0.505
Saint Kitts and Nevis	384	734	0.523
Saint Lucia	223	685	0.326
Saint Vincent and the Grenadines	281	648	0.434
Samoa	749	1126	0.665
Sao Tome and Principe	536	619	0.866
Saudi Arabia	435	769	0.566
Senegal	449	587	0.765
Serbia	292	737	0.396
Sierra Leone	436	489	0.892
Slovakia	570	941	0.606
Slovenia	521	1011	0.515
Solomon Islands	335	431	0.777
South Africa	340	681	0.499
South Korea	488	722	0.676
Spain	793	1323	0.599
Sri Lanka	352	398	0.884
Sudan	243	628	0.387
Suriname	364	602	0.605
Sweden	637	1135	0.561
Switzerland	709	1375	0.516
Tajikistan	241	475	0.507
Tanzania	218	332	0.657
Thailand	276	490	0.563
Togo	288	411	0.701
Trinidad and Tobago	440	705	0.624
Tunisia	565	797	0.709
Turkey	644	928	0.694
Turkmenistan	265	660	0.402
Uganda	246	359	0.685
Ukraine	386	755	0.511
United Arab Emirates	418	865	0.483
United Kingdom	637	1258	0.506
United States	811	1374	0.590
Uruguay	325	879	0.370
Uzbekistan	316	616	0.513
Vanuatu	702	880	0.798
Venezuela	399	658	0.606
Vietnam	150	423	0.355
Yemen	215	381	0.564
Zambia	153	309	0.495
Zimbabwe	314	476	0.660
Overall averages	397	717	0.556

The mean ratio of the 169 countries was 0.556. We used this value as the ratio for the 26 countries that did not have data. To derive the added SFA, added PUFA, and added TFA for each country, we multiplied the SFA, PUFA, and TFA of that country by their respective ratios of the added fats and oils/total fat (KC/d).

Statistical methods

To determine the strengths of the risk factor correlations with NCDs, we utilized Pearson correlation coefficients: r, 95% confidence intervals (CIs), and p values. We did this for the entire analysis dataset and subgroups including continents, countries, and sociodemographic index quartiles.

The EAT-Lancet Commission authors [2] considered 0-400 KC/d of animal foods as optimal for human health and global climate. For our comparison, we began with finding the 1000 cohorts (500 pairs of males and females), representing about one billion people 15-69 years old with the lowest NCDs (mean male/female {m/f}). From these low-NCD cohorts, we obtained the subsets with mean animal-sourced food consumption of <400 KC/d and ≥400 KC/d. From this start, we defined low and high animal food subsets as follows: (1) Low animal food subset=animal food seven of <400 KC/d of the lowest 1000 NCD cohorts (mean KC/d m/f)+all cohorts with animal food seven (mean m/f KC/d)<the animal food seven of the lowest NCD cohorts’ country with the lowest animal food seven (e.g., Kenya). (2) High animal food subset=all 1000 lowest NCD cohorts+all cohorts with animal food seven of ≥400 KC/d.

With these low animal food and high animal food subsets, we derived multiple regression formulas from NCDs (dependent variable) versus dietary and other risk factors (independent variables). See Appendices for the detailed methodology of deriving multiple regression formulas with risk factor coefficients equated to their population-attributable risk percents (PAR%s).

We also developed a methodology for estimating the optimal dietary risk factor (KC/d) range upper and lower boundaries to minimize NCDs (see Appendices). We used SAS OnDemand for Academics software 9.4 (SAS Institute, Cary, NC) for the data analysis.

## Results

Table 5 shows the basic statistics for GBD dietary and other risk factors and NCD data from 195 countries.

**Table 5:** Worldwide risk factor and NCD basic statistics NCD, noncommunicable disease; SD, standard deviation; CI, confidence interval; SFA, saturated fatty acid; PUFA, polyunsaturated fatty acid; TFA, trans fatty acid; METs, metabolic equivalent of tasks; PM_0.25_, particulate matter 0.25; BMI, body mass index; LDL, low-density lipoprotein; BP, blood pressure; KC/d, kilocalories/day

NCD deaths/100000/year versus risk factors, n=7846 cohorts	Mean	SD	Minimum	Maximum	r	95% CI low	95% CI high	P
NCD deaths/100000/year	1428	462.97	423.80	4321				
Processed meat, KC/d	5.33	9.72	0.20	68.77	-0.147	-0.169	-0.126	<0.0001
Red meat, KC/d	50.27	45.13	3.21	235.95	-0.118	-0.140	-0.096	<0.0001
Fish, KC/d	9.99	36.52	0.40	370.36	-0.228	-0.249	-0.207	<0.0001
Milk, KC/d	25.04	27.05	1.06	146.82	-0.162	-0.184	-0.141	<0.0001
Poultry (KC/d) available	39.94	39.88	1.40	289.96	-0.293	-0.313	-0.272	<0.0001
Eggs (KC/d) available	18.36	13.16	1.05	63.43	-0.335	-0.354	-0.315	<0.0001
Added SFA, KC/d	105.73	40.86	16.78	342.63	-0.083	-0.105	-0.061	<0.0001
Added PUFA, KC/d	44.40	42.30	1.32	229.82	-0.226	-0.247	-0.205	<0.0001
Added TFA, KC/d	7.45	7.43	0.75	38.98	-0.090	-0.111	-0.068	<0.0001
Alcohol, KC/d	81.03	57.33	4.25	429.81	0.129	0.107	0.151	<0.0001
Sugary beverages, KC/d	298.36	152.38	72.91	1472.00	0.110	0.088	0.132	<0.0001
Potatoes (KC/d) available	84.46	82.15	3.49	666.70	0.047	0.025	0.069	<0.0001
Sweet potatoes (KC/d) available	24.60	38.59	0.02	438.25	-0.085	-0.107	-0.063	<0.0001
Corn (KC/d) available	36.25	51.54	0.16	351.50	-0.037	-0.059	-0.015	0.0011
Fruits, KC/d	40.21	22.50	3.58	161.39	-0.424	-0.442	-0.406	<0.0001
Vegetables, KC/d	79.76	43.12	9.48	304.17	-0.141	-0.163	-0.120	<0.0001
Nuts and seeds, KC/d	8.41	8.36	0.05	102.99	-0.232	-0.253	-0.211	<0.0001
Whole grains, KC/d	55.65	30.93	1.14	235.10	-0.128	-0.149	-0.106	<0.0001
Legumes, KC/d	51.74	32.23	0.51	194.70	0.097	0.075	0.119	<0.0001
Rice (KC/d) available	152.00	130.75	2.33	547.15	0.052	0.030	0.075	<0.0001
Animal food seven	254.66	153.44	51.45	794.80	-0.254	-0.274	-0.233	<0.0001
Healthy plant food seven	304.76	92.66	62.20	748.17	-0.337	-0.357	-0.318	<0.0001
Total KC/d available	2574	418.33	1579	3898	-0.264	-0.284	-0.243	<0.0001
Vitamin A deficiency in children/100000/year	23205	10939	1267	50969	0.242	0.221	0.263	<0.0001
Sodium, gram/day	4.45	2.34	1.33	9.21	-0.139	-0.161	-0.118	<0.0001
Calcium, gram/day	0.301	0.179	0.081	1.044	-0.141	-0.163	-0.120	<0.0001
Dietary fiber, gram/day	9.21	3.15	2.72	22.68	0.030	0.008	0.052	0.0072
Physical activity METs	4714	1368	1609	7669	0.267	0.246	0.287	<0.0001
Child underweight of >2 SD	0.186	0.171	0.004	0.535	0.325	0.305	0.345	<0.0001
Stop breast feeding in <6 months	0.119	0.055	0.016	0.242	-0.325	-0.344	-0.305	<0.0001
Ambient pollution, PM_2.5_	44.73	26.46	4.38	95.54	0.214	0.192	0.235	<0.0001
Smoking rate (0-1)	0.205	0.176	0.003	0.640	0.380	0.361	0.399	<0.0001
Secondhand smoking (0-1)	0.376	0.155	0.164	0.796	-0.339	-0.359	-0.320	<0.0001
Sublingual tobacco rate (0-1)	0.068	0.095	0.001	0.419	0.319	0.299	0.339	<0.0001
Blood lead level, mcg/dL	5.01	1.01	1.22	8.37	0.231	0.210	0.252	<0.0001
Household air pollution (0-1)	0.482	0.325	0.000	0.996	0.272	0.251	0.292	<0.0001
Kidney disease stage III (0-1)	0.056	0.028	0.015	0.154	0.032	0.010	0.054	0.0041
BMI, kg/m^2^	21.768	2.291	17.949	29.386	-0.304	-0.324	-0.284	<0.0001
LDL cholesterol, mmol/L	2.35	0.40	1.27	3.25	-0.339	-0.358	-0.319	<0.0001
Fasting plasma glucose, mmol/L	4.30	0.35	3.32	5.58	-0.135	-0.156	-0.113	<0.0001
Systolic BP, mm Hg	133.91	4.32	123.41	147.89	0.063	0.041	0.085	<0.0001
Sociodemographic index (0-1)	0.543	0.174	0.112	0.896	-0.340	-0.360	-0.321	<0.0001
Sex: male, one; female, two	1.50	0.50	1.00	2.00	-0.512	-0.528	-0.495	<0.0001

There were 23 dietary risk factors potentially relating to NCDs, including two risk factor combinations, six dietary covariates, and total available KC/d. Table 5 also includes 20 non-dietary risk factors that we screened for significant PAR%s for NCDs.

Table 6 shows the 500 pairs (mean males/females) of cohorts (n=1000 cohorts, representing about one billion people) with the lowest NCDs and the 500 pairs of cohorts with the highest NCDs.

**Table 6:** The 500 pairs of cohorts (1000 cohorts) with the lowest and highest NCDs NCD, noncommunicable disease; SD, standard deviation; SFA, saturated fatty acid; PUFA, polyunsaturated fatty acid; TFA, trans fatty acid; METs, metabolic equivalent of tasks; PM_0.25_, particulate matter 0.25; BMI, body mass index; LDL, low-density lipoprotein; BP, blood pressure; KC/d, kilocalories/day; m/f, male/female

Low-NCD and high-NCD subsets compared	NCD mean m/f of ≤1070.23 deaths/100000/year; dietary risk factors (KC/d) are mean of m/f; n=500 cohort pairs				NCD mean m/f of >1763.60 deaths/100000/year; dietary risk factors (KC/d) are means of m/f; n=500 cohort pairs			
Dietary and other risk factors for NCDs, KC/d for all foods, and mean of males and females for all	Mean	SD	Minimum	Maximum	Mean	SD	Minimum	Maximum
NCD deaths/100000/year	925.24	113.11	634.58	1070	2080	313.65	1764	3521
Processed meat, KC/d	12.36	11.34	1.01	53.09	3.94	5.54	0.25	54.02
Red meat, KC/d	82.10	44.78	14.93	171.01	40.00	34.27	8.19	158.86
Fish, KC/d	48.94	92.40	2.89	337.58	4.42	3.33	0.44	18.68
Milk, KC/d	53.99	32.20	15.09	135.11	24.83	20.33	1.08	98.58
Poultry (KC/d) available	76.69	35.24	5.75	184.45	30.57	23.57	1.40	155.02
Eggs (KC/d) available	28.48	12.53	5.78	63.43	13.89	12.22	1.05	41.64
Added SFA, KC/d	139.04	43.82	80.82	260.53	102.65	37.43	23.72	316.45
Added PUFA, KC/d	82.99	37.38	17.95	209.68	28.41	24.62	2.04	195.24
Added TFA, KC/d	10.16	9.31	2.21	36.96	4.68	3.23	1.08	25.46
Alcohol, KC/d	103.22	61.00	16.89	241.47	60.95	39.88	6.21	259.17
Sugary beverages, KC/d	350.35	246.77	92.46	1392.00	293.61	59.39	152.38	641.82
Potatoes (KC/d) available	83.07	45.26	8.30	287.77	119.81	120.96	4.04	666.70
Sweet potatoes (KC/d) available	4.68	7.14	0.03	33.57	13.77	35.50	0.02	438.25
Corn (KC/d) available	34.70	50.06	1.46	227.31	40.39	58.11	0.20	351.50
Fruits, KC/d	63.78	17.97	23.15	107.07	25.94	14.39	3.84	101.01
Vegetables, KC/d	106.05	47.88	9.62	221.12	61.45	42.13	14.93	146.75
Nuts and seeds, KC/d	14.64	11.63	0.27	44.19	4.82	3.96	0.05	48.43
Whole grains, KC/d	50.05	32.00	1.64	150.57	36.38	33.89	1.39	140.95
Legumes, KC/d	50.14	27.47	3.27	123.96	39.52	29.72	0.69	180.77
Rice (KC/d) available	56.20	50.15	2.93	159.66	94.32	149.39	2.33	547.15
Animal food seven, KC/d	441.61	150.98	149.45	737.21	220.30	113.18	62.51	680.49
Healthy plant food seven, KC/d	372.41	79.64	203.52	662.45	210.34	82.78	62.20	606.18
Total KC/d available	2959	401	1948	3572	2392	490	1579	3481
Vitamin A deficiency in children/100000/year	14044	8918	1368	44100	24790	13443	1718	50969
Sodium, gram/day	3.67	1.15	1.33	6.70	3.28	0.90	1.33	6.47
Calcium, gram/day	0.49	0.17	0.19	1.04	0.30	0.17	0.08	0.86
Dietary fiber, gram/day	10.82	2.32	5.41	18.15	9.18	2.97	2.72	20.67
Physical activity METs	3389	1019	1609	7607	4944	1439	1708	7669
Child underweight of >2 SD	0.05	0.04	0.00	0.24	0.21	0.15	0.00	0.41
Stop breast feeding of <6 months	0.17	0.04	0.07	0.23	0.11	0.06	0.03	0.22
Ambient pollution, PM_0.25_	19.92	14.45	4.38	81.45	34.60	17.48	6.78	63.87
Smoking rate (0-1)	0.21	0.11	0.01	0.46	0.21	0.18	0.00	0.63
Secondhand smoking (0-1)	0.33	0.09	0.16	0.65	0.36	0.16	0.16	0.78
Sublingual tobacco rate (0-1)	0.01	0.02	0.00	0.11	0.05	0.07	0.00	0.21
Blood lead level, mcg/dL	4.23	1.15	1.22	7.20	4.49	1.19	1.92	8.37
Household air pollution (0-1)	0.10	0.17	0.00	0.84	0.52	0.37	0.00	1.00
Kidney disease stage III (0-1)	0.05	0.03	0.02	0.12	0.07	0.02	0.03	0.14
BMI, kg/m^2^	23.76	1.49	19.61	28.57	21.65	2.36	17.95	27.73
LDL cholesterol, mmol/L	2.74	0.30	1.60	3.25	2.28	0.50	1.34	3.20
Fasting plasma glucose, mmol/L	4.49	0.30	3.54	5.58	4.25	0.47	3.38	5.18
Systolic BP, mm Hg	134.07	4.05	123.41	144.35	135.71	5.38	123.74	147.89
Sociodemographic index (0-1)	0.72	0.14	0.35	0.89	0.46	0.22	0.11	0.87
Sex: male, one; female, two	1.50	0.50	1.00	2.00	1.50	0.50	1.00	2.00

The animal food seven (KC/d) of the lowest NCD cohorts was about double the animal food seven (KC/d) from the highest NCD cohorts. From the lowest NCD subset in Table 6, Table 7 reports the cohort pairs that had animal food seven of <400 KC/d (416 cohorts) and animal food seven of ≥400 KC/d (584 cohorts).

**Table 7:** The 1000 cohorts with lowest NCDs and subsets with animal foods of >400 KC/d NCD, noncommunicable disease; SD, standard deviation; SFA, saturated fatty acid; PUFA, polyunsaturated fatty acid; TFA, trans fatty acid; METs, metabolic equivalent of tasks; PM_0.25_, particulate matter 0.25; BMI, body mass index; LDLc, low-density lipoprotein cholesterol; FPG, fasting plasma glucose; SBP, systolic blood pressure; KC/d, kilocalories/day; SDI, sociodemographic index

	Animal food seven of <400 KC/d (n=418 cohorts)				Animal food seven of >400 KC/d (n=584 cohorts)			
Dietary and other risk factors for NCD expressed as means of males and females from the lowest NCD 500 pairs of cohorts (n=1000 cohorts)	Mean	SD	Minimum	Maximum	Mean	SD	Minimum	Maximum
NCD deaths/100000/year	948.47	87.64	713.37	1064	908.70	125.68	634.58	1070
Processed meat, KC/d	2.99	2.93	1.01	13.26	19.04	10.34	1.89	53.09
Red meat, KC/d	43.95	24.28	14.93	108.84	109.27	35.18	51.90	171.01
Fish, KC/d	6.62	2.47	2.89	11.52	79.09	111.53	6.75	337.58
Milk, KC/d	28.38	8.36	15.34	76.05	72.24	30.42	15.09	135.11
Poultry, KC/d	69.19	36.14	5.75	143.64	82.02	33.62	34.05	184.45
Eggs, KC/d	18.98	7.11	5.78	38.45	35.26	11.08	17.34	63.43
Added SFA, KC/d	112.11	25.72	80.82	173.02	158.22	43.97	82.02	260.53
Added PUFA, KC/d	70.50	30.23	17.95	142.96	91.90	39.41	44.10	209.68
Added TFA, KC/d	11.40	9.87	2.21	36.96	9.27	8.78	2.86	36.25
Alcohol, KC/d	51.29	36.72	16.89	177.64	140.21	46.06	16.97	241.47
Sugary beverages, KC/d	520.29	283.48	98.34	1392.00	229.30	108.91	92.46	596.36
Potatoes, KC/d	86.51	56.89	17.26	287.77	80.61	34.53	8.30	143.58
Sweet potatoes, KC/d	5.61	9.21	0.16	33.57	4.02	5.09	0.03	13.81
Corn, KC/d	62.24	67.72	2.37	227.31	15.08	10.20	1.46	40.93
Fruits, KC/d	63.03	20.74	23.15	107.07	64.31	15.70	35.89	96.69
Vegetables, KC/d	78.32	51.12	9.62	189.32	125.79	33.61	44.10	221.12
Nuts/seeds, KC/d	7.29	10.05	0.27	31.59	19.88	9.68	0.88	44.19
Whole grains, KC/d	55.11	38.68	2.30	150.57	46.44	25.66	1.64	86.76
Legumes, KC/d	58.67	28.11	21.99	123.96	44.05	25.32	3.27	122.99
Rice, KC/d	69.84	44.37	2.93	159.66	46.49	51.77	5.95	137.99
Animal food seven, KC/d	282.22	77.62	149.45	396.61	555.14	61.09	404.36	737.21
Healthy plant seven, KC/d	338.53	74.06	243.65	467.13	396.39	72.23	207.63	630.85
Total KC/d	2741	353	1948	3193	3114	359	2456	3572
Vitamin A deficiency in children/100000/year	17616	9137	1368	44100	11499	7826	1400	28081
Sodium, gram/day	3.04	0.56	2.06	5.93	4.12	1.21	2.64	6.56
Calcium, gram/day	0.34	0.06	0.20	0.73	0.599	0.139	0.362	1.004
Fiber, gram/day	10.85	2.46	5.90	16.32	10.80	1.97	7.05	16.62
Physical activity METs	3449	1217	2170	7496	3347	520.11	2085	5016
Child underweight of >2 SD	0.080	0.045	0.014	0.205	0.020	0.016	0.006	0.057
Stop breast feeding in <6 months	0.146	0.044	0.072	0.226	0.189	0.017	0.134	0.217
Ambient pollution, PM_0.25_	29.40	17.30	8.87	81.45	13.16	5.91	4.38	61.39
Smoking rate (0-1)	0.139	0.051	0.050	0.282	0.252	0.043	0.110	0.382
Secondhand smoke (0-1)	0.332	0.073	0.178	0.490	0.332	0.043	0.218	0.451
Sublingual tobacco (0-1)	0.021	0.025	0.002	0.058	0.009	0.009	0.001	0.057
Blood lead, mcg/dL	5.13	0.72	3.76	6.57	3.59	0.77	1.45	5.99
Household air pollution (0-1)	0.211	0.214	0.006	0.829	0.013	0.027	0.001	0.201
Kidney disease III (0-1)	0.072	0.016	0.032	0.097	0.032	0.008	0.019	0.086
BMI kg/m^2^	23.63	1.22	20.20	25.98	23.86	1.54	21.44	28.22
LDLc, mmol/L	2.46	0.24	1.69	3.02	2.93	0.13	2.64	3.24
FPG, mmol/L	4.28	0.29	3.62	4.99	4.64	0.17	4.18	5.30
SBP, mm Hg	132.31	3.65	125.19	143.43	135.33	3.31	127.15	140.44
SDI (0-1)	0.564	0.074	0.351	0.792	0.826	0.049	0.490	0.89
Sex: male, one; female, two	1.50	0.50	1.00	2.00	1.50	0.50	1.00	2.00

This breakdown will facilitate the comparisons of the EAT-Lancet Commission’s Planetary Health Diet recommendations with GBD data analysis.

Table 8 and Table 9 list the low-NCD countries in Table 7, distinguishing the low-NCD countries or subnational states/regions with mean animal food seven of <400 KC/d and animal food seven of ≥400 KC/d.

**Table 8:** Twenty countries from Table 7 with risk factors and NCDs with animal food seven of <400 KC/d NCD, noncommunicable disease; KC/d, kilocalories/day; m/f, male/female

Twenty countries with NCDs of <1070.22659 deaths/100000/year and animal food seven of <400 KC/d, n=416 cohorts, means m/f	NCDs	Non-cancer NCDs	Cancer NCDs	Animal food seven	Healthy plant food seven	Sodium, gram/day	Smoking prevalence
Albania	885.06	483.58	231.47	275.91	284.12	4.16	0.221
Singapore	1015.04	381.20	232.63	372.50	389.21	5.93	0.146
Cuba	1049.46	522.49	268.81	244.57	467.18	2.26	0.282
Dominican Republic	926.28	624.60	160.32	364.01	408.38	2.18	0.128
Ecuador	913.50	471.12	178.12	364.22	272.90	3.16	0.080
Peru	843.18	363.04	188.20	180.25	243.68	3.14	0.075
Colombia	1040.29	454.28	193.10	312.31	275.09	3.69	0.132
Costa Rica	887.87	412.25	205.02	356.47	331.93	2.96	0.146
Nicaragua	940.59	564.36	133.81	196.59	255.97	2.65	0.085
Panama	746.05	379.22	164.02	346.10	306.89	2.97	0.073
Paraguay	970.03	584.60	184.19	326.18	376.37	3.07	0.155
Algeria	1002.30	619.12	120.86	200.13	273.92	3.05	0.202
Saudi Arabia	863.41	612.85	115.45	363.60	277.95	3.42	0.100
Tunisia	976.32	613.06	162.45	229.04	434.41	3.05	0.233
Cape Verde	969.89	515.35	228.24	234.62	318.55	2.35	0.078
Mexico (48/130 cohorts)	953.2	551.68	145.00	310.71	395.75	2.58	0.117
South Africa	1016.27	806.94	221.62	324.79	273.86	2.46	0.219
Brazil	982.78	567.72	182.62	380.95	382.50	3.06	0.137
Kenya	957.96	578.62	151.64	149.62	297.12	2.06	0.123
Iran	866.98	513.94	149.52	203.35	450.72	3.09	0.143

**Table 9:** Countries from Table 7 with animal food seven of ≥400 KC/d NCD, noncommunicable disease; KC/d, kilocalories/day; m/f, male/female

Twenty-four countries with NCDs of <1070.22659 deaths/100000/year and animal food seven of ≥400 KC/d, n=584 cohorts, means m/f	NCDs	Non-cancer NCDs	Cancer NCDs	Animal food seven	Healthy plant food seven	Sodium, gram/day	Smoking prevalence
Taiwan	1070.23	410.56	300.82	411.76	631.05	3.23	0.208
Australia	975.05	285.91	285.53	642.03	305.88	2.85	0.194
Andorra	786.88	258.70	271.69	637.21	442.05	3.68	0.259
Belgium	1061.43	344.36	367.89	569.98	414.03	3.08	0.257
Cyprus	951.47	421.10	259.41	440.18	333.85	3.32	0.329
France	962.34	260.13	361.89	555.89	285.74	3.21	0.289
Greece	885.96	379.55	311.71	496.14	469.85	3.34	0.382
Iceland	871.96	251.33	287.52	451.17	207.69	2.89	0.217
Israel	989.51	320.64	283.39	529.95	554.77	3.21	0.226
Italy	995.59	289.11	338.60	511.83	413.29	3.56	0.248
Malta	999.14	371.04	270.49	408.66	377.57	3.43	0.244
Netherlands	1033.16	310.08	378.65	525.89	360.84	3.06	0.261
Spain	929.45	304.85	311.74	537.27	430.07	3.20	0.306
Switzerland	824.61	239.36	283.52	509.91	276.50	3.54	0.267
Canada	993.48	303.04	331.29	583.25	389.22	3.71	0.206
Kuwait	713.09	431.02	108.03	497.31	466.80	3.79	0.176
Northern Mariana Islands	1000.03	565.51	191.22	539.42	326.42	3.36	0.195
United Kingdom (20/66 cohorts)	1016.00	328.16	311.15	469.28	298.02	2.66	0.228
Japan (158/158 cohorts)	725.61	236.47	288.29	576.46	428.03	6.01	0.268
United States (50/336 cohorts)	1005.19	389.19	294.71	649.01	458.32	4.06	0.189
Brazil	1002.00	571.10	186.32	408.08	440.03	3.18	0.156
Sweden	896.82	307.74	279.60	540.12	265.72	3.93	0.176
New Zealand	1069.71	321.82	315.75	626.76	369.82	3.98	0.201
Norway	987.25	306.75	308.91	493.52	364.15	3.69	0.264

For low-NCD countries or subnational states/regions with mean animal food seven of <400 KC/d, non-cancer NCDs exceeded cancer NCDs by about 3.2-fold (i.e., non-cancer NCDs=538.47 deaths/100000/year, and cancer NCDs=166.59 deaths/100000/year; 538.47/166.59=3.23). Conversely, for countries or subnational states/regions with mean animal food seven of ≥400 KC/d overall, the deaths from cancer NCDs slightly exceeded early deaths from non-cancer NCDs (i.e., non-cancer NCDs=295.61 deaths/100000/year, and cancer NCDs=310.28).

Table 10 shows scenarios of data from 12 continents, countries, and SDI quadrants that illustrate the diverse relationships of animal food seven with NCDs in different subsets of the global analysis dataset. For example, animal food seven positively correlated with NCDs in five subsets, negatively correlated with NCDs in six subsets, and has no significant correlation in one.

**Table 10:** Scenarios of continents, countries, and SDI quadrants NCD, noncommunicable disease; SDI, sociodemographic index; SD, standard deviation; CI, confidence interval; SFA, saturated fatty acid; PUFA, polyunsaturated fatty acid; TFA, trans fatty acid; NA, not available

NCDs correlated with dietary risk factors for continents, countries, and SDI quadrants								
Africa, n=1682 cohorts	Mean	SD	Minimum	Maximum	r	95% CI low	95% CI high	P
NCD deaths/100000/year	1509	475.77	609	4321				
Processed meat	1.35	0.57	0.3	4.13	-0.299	-0.342	-0.255	<0.0001
Red meat	20.96	11.14	4.28	78.22	0.031	-0.017	0.079	0.2053
Fish	3.14	2.59	0.4	20.64	-0.217	-0.262	-0.171	<0.0001
Milk	12.76	10.82	1.2	66.95	-0.138	-0.185	-0.091	<0.0001
Poultry	27.78	35.79	1.4	272.54	-0.25	-0.294	-0.204	<0.0001
Eggs	7.72	5.44	1.05	30.1	-0.406	-0.445	-0.365	<0.0001
Added SFA	97.27	35.79	18.28	242.77	-0.399	-0.439	-0.358	<0.0001
Added PUFA	33.04	32.38	1.45	197.91	-0.262	-0.306	-0.217	<0.0001
Added TFA	6.25	8.32	0.69	37.01	-0.097	-0.144	-0.05	<0.0001
Alcohol	53.33	42.38	5.92	316.45	0.12	0.073	0.167	<0.0001
Sugary beverages	267.52	57.43	171.24	442.06	0.119	0.071	0.165	<0.0001
Potatoes	144.11	142.33	3.49	666.7	-0.067	-0.115	-0.019	0.0059
Sweet potatoes	41.82	59.69	0.04	283.45	-0.153	-0.2	-0.106	<0.0001
Corn	73.06	76.57	0.26	351.5	0.024	-0.024	0.071	0.3299
Fruits	41.36	28.09	3.58	129.49	-0.321	-0.363	-0.277	<0.0001
Vegetables	76.56	65.3	10.83	304.17	-0.188	-0.233	-0.141	<0.0001
Nuts and seeds	10.39	10.39	0.47	102.99	-0.311	-0.353	-0.267	<0.0001
Whole grains	43.48	30.23	1.6	112.17	-0.078	-0.126	-0.031	0.0013
Legumes	65.94	35.49	10.67	194.7	-0.058	-0.105	-0.01	0.0177
Rice	54.56	59.45	2.33	345.85	-0.041	-0.089	0.006	0.0896
Animal food seven	170.98	71.26	54.03	610.4	-0.383	-0.423	-0.342	<0.0001
Healthy plant seven	312.6	115.21	83.01	737.86	-0.404	-0.443	-0.363	<0.0001
Asia, n=4188 cohorts	Mean	SD	Minimum	Maximum	r	95% CI low	95% CI high	P
NCD deaths/100000/year	1437	352.91	423.8	2956				
Processed meat	2.29	3.51	0.2	27.62	-0.393	-0.418	-0.367	<0.0001
Red meat	38.46	33.76	3.21	129.83	-0.244	-0.272	-0.215	<0.0001
Fish	12.57	49.63	0.62	370.36	-0.389	-0.414	-0.363	<0.0001
Milk	12.57	8.52	1.06	35.4	0.147	0.118	0.177	<0.0001
Poultry	23.81	19.89	3.34	172.27	-0.447	-0.471	-0.423	<0.0001
Eggs	18.63	14.38	3.56	63.43	-0.52	-0.542	-0.498	<0.0001
Added SFA	89.72	24.06	23.7	255.96	0.046	0.016	0.077	0.0027
Added PUFA	27.59	21.09	2.01	212.01	-0.317	-0.344	-0.289	<0.0001
Added TFA	5.82	3.88	1.41	13.05	0.283	0.255	0.311	<0.0001
Alcohol	80.14	50.15	5.77	219.57	0.191	0.162	0.22	<0.0001
Sugary beverages	263.91	66.59	72.91	397.35	0.705	0.689	0.72	<0.0001
Potatoes	50.77	20.48	8.3	111.01	-0.057	-0.087	-0.026	0.0002
Sweet potatoes	27.61	29.88	0.16	68.96	-0.283	-0.31	-0.254	<0.0001
Corn	20.9	18.54	0.54	198.21	-0.083	-0.113	-0.053	<0.0001
Fruits	30.67	13.19	10.16	80.76	-0.494	-0.517	-0.471	<0.0001
Vegetables	75.2	29.99	17.69	188.72	-0.334	-0.36	-0.306	<0.0001
Nuts and seeds	5.52	3.52	1.1	49.94	-0.069	-0.099	-0.038	<0.0001
Whole grains	63.13	27.81	4.59	156.91	-0.064	-0.094	-0.034	<0.0001
Legumes	51.88	28.83	13.64	133.26	0.37	0.344	0.396	<0.0001
Rice	248.06	100.11	47.64	547.15	0.123	0.093	0.153	<0.0001
Animal food seven	198.05	95.06	70.88	786.29	-0.452	-0.475	-0.427	<0.0001
Healthy plant seven	281.59	67.54	111.34	644.64	-0.341	-0.367	-0.314	<0.0001
South America, n=880 cohorts	Mean	SD	Minimum	Maximum	r	95% CI low	95% CI high	P
NCD deaths/100000/year	1500	766.41	513.13	3193				
Processed meat	13.6	9.47	1.29	55.2	-0.137	-0.201	-0.071	<0.0001
Red meat	104.31	38.9	16.67	235.95	-0.019	-0.085	0.047	0.5738
Fish	8.79	2.44	1.66	17.77	-0.044	-0.11	0.022	0.1891
Milk	72.36	25.15	17.52	146.82	-0.545	-0.589	-0.496	<0.0001
Poultry	56.8	23.84	2.7	184.45	-0.203	-0.266	-0.139	<0.0001
Eggs	28.68	7.13	3.06	41.64	0.031	-0.035	0.097	0.36
Added SFA	148.56	37.04	54.63	242.59	-0.508	-0.555	-0.457	<0.0001
Added PUFA	68.87	35.21	9.08	162.99	-0.438	-0.49	-0.383	<0.0001
Added TFA	6.39	3.7	1.41	25.22	-0.295	-0.354	-0.233	<0.0001
Alcohol	94.68	42.36	8.51	290.45	0.265	0.202	0.325	<0.0001
Sugary beverages	318.35	72.08	148.41	625.68	0.567	0.521	0.61	<0.0001
Potatoes	130.82	52.4	46.14	286.52	0.462	0.409	0.513	<0.0001
Sweet potatoes	0.26	0.29	0.02	1.78	-0.312	-0.37	-0.251	<0.0001
Corn	12.11	18.11	0.16	157.04	0	-0.066	0.066	0.9948
Fruits	50.64	21.85	9.64	128.61	-0.653	-0.689	-0.613	<0.0001
Vegetables	113.86	33.3	29.69	228.75	0.055	-0.011	0.121	0.1035
Nuts and seeds	14.85	10.28	2.34	45.28	-0.55	-0.595	-0.502	<0.0001
Whole grains	30.35	22.16	1.14	92.45	-0.162	-0.226	-0.097	<0.0001
Legumes	17.96	11.09	0.51	56.78	-0.223	-0.285	-0.16	<0.0001
Rice	10.57	5.67	3.14	33.49	0.011	-0.055	0.077	0.7354
Animal food seven	433.11	110.86	145.49	673.8	-0.354	-0.411	-0.295	<0.0001
Healthy plant seven	296.79	82.03	63.27	561.31	-0.484	-0.533	-0.431	<0.0001
North America, n=558 cohorts	Mean	SD	Minimum	Maximum	r	95% CI low	95% CI high	P
NCD deaths/100000/year	1187	347.01	687.38	2633				
Processed meat	28.55	17.67	0.83	68.77	0.286	0.208	0.36	<0.0001
Red meat	110.41	47.48	10.13	207.84	0.276	0.197	0.351	<0.0001
Fish	13.11	4.02	1.77	21.62	0.033	-0.05	0.115	0.4403
Milk	69.37	27.57	4.93	101.23	-0.052	-0.135	0.031	0.2161
Poultry	123.6	37.78	17.98	269.1	-0.012	-0.095	0.071	0.7777
Eggs	30.24	6.11	2.2	40.6	-0.314	-0.387	-0.237	<0.0001
Added SFA	160.56	40.56	70.24	258.1	-0.109	-0.19	-0.026	0.0099
Added PUFA	126.51	49.96	21.08	186.09	0.04	-0.043	0.123	0.3458
Added TFA	25.93	9.12	1.98	42.56	-0.462	-0.525	-0.394	<0.0001
Alcohol	167.04	76.22	4.25	296.7	0.416	0.345	0.483	<0.0001
Sugary beverages	371.73	332.57	130.99	1472	0.016	-0.067	0.099	0.7125
Potatoes	68.01	29.69	17.26	123.42	0.061	-0.022	0.143	0.1494
Sweet potatoes	5.48	15.78	0.54	104.54	0.432	0.362	0.497	<0.0001
Corn	63.44	81.28	2.13	236.88	-0.089	-0.17	-0.006	0.0364
Fruits	69.43	10.86	34.65	143.26	-0.465	-0.528	-0.397	<0.0001
Vegetables	97.15	35.95	25.62	224.91	-0.095	-0.177	-0.012	0.0248
Nuts and seeds	18.23	9.98	0.56	38.04	0.024	-0.059	0.107	0.5749
Whole grains	75.01	39.36	21.06	235.1	-0.107	-0.188	-0.024	0.0114
Legumes	50.64	22.16	9.98	133.5	0.118	0.035	0.199	0.0053
Rice	22.53	33.12	11.2	246.08	0.203	0.122	0.281	<0.0001
Animal food seven	535.85	157.3	135.82	734.89	0.048	0.064	-0.019	0.1461
Healthy plant seven	442.46	53.69	227.6	604.11	-0.019	-0.102	0.064	0.6551
Oceania, n=54 cohorts	Mean	SD	Minimum	Maximum	r	95% CI low	95% CI high	P
NCD deaths/100000/year	1782	1031	704.17	4015				
Processed meat	5.7	4.61	0.46	11.04	-0.729	-0.834	-0.573	<0.0001
Red meat	106.56	69.22	15.85	198.7	-0.645	-0.778	-0.456	<0.0001
Fish	8.81	5.62	1.44	15.22	-0.789	-0.873	-0.661	<0.0001
Milk	55.73	44.86	2.62	97.95	-0.79	-0.873	-0.662	<0.0001
Poultry	78.31	43.94	6.81	118.26	-0.842	-0.906	-0.741	<0.0001
Eggs	15.89	7.06	7	29.82	-0.678	-0.8	-0.501	<0.0001
Added SFA	220.54	32.46	179.39	316.82	-0.225	-0.465	0.046	0.0993
Added PUFA	41.33	24.13	14.52	130.67	-0.407	-0.609	-0.157	0.0018
Added TFA	8.98	4.69	2.85	14.5	-0.85	-0.911	-0.754	<0.0001
Alcohol	131.27	75.41	18.8	340.37	0.81	0.692	0.885	<0.0001
Sugary beverages	237.01	64.24	158.38	385.37	0.825	0.716	0.895	<0.0001
Potatoes	62.12	37.95	4.04	98.69	-0.75	-0.848	-0.604	<0.0001
Sweet potatoes	24.27	82.36	0.94	438.25	0.178	-0.095	0.425	0.1953
Corn	4.26	3.11	0.2	7.39	-0.791	-0.874	-0.663	<0.0001
Fruits	47.49	15.72	19.2	72.43	-0.802	-0.881	-0.681	<0.0001
Vegetables	67.73	35.37	16.68	125.96	-0.718	-0.827	-0.558	<0.0001
Nuts and seeds	12.94	12.2	0.05	26.39	-0.776	-0.864	-0.642	<0.0001
Whole grains	48.19	15.11	25.91	64.31	-0.809	-0.885	-0.691	<0.0001
Legumes	24.8	8.76	15.74	57.3	-0.022	-0.288	0.247	0.8734
Rice	59.9	49.34	14.45	159.66	0.757	0.614	0.852	<0.0001
Animal food seven	491.53	173.79	238.8	679.17	-0.789	-0.872	-0.66	<0.0001
Healthy plant seven	266.75	103.9	134.63	594.28	-0.528	-0.697	-0.304	<0.0001
Europe, n=468 cohorts	Mean	SD	Minimum	Maximum	r	95% CI low	95% CI high	P
NCD deaths/100000/year	1171	330.12	644.73	2232				
Processed meat	3.57	3.47	0.78	20.97	0.135	0.045	0.223	0.0033
Red meat	80.46	44.21	12.03	192.51	0.67	0.616	0.717	<0.0001
Fish	9.93	3.9	3.52	27.73	0.596	0.534	0.652	<0.0001
Milk	34.82	12.97	9.54	103.76	0.265	0.178	0.347	<0.0001
Poultry	86.79	22.06	8.89	127.44	0.293	0.208	0.373	<0.0001
Eggs	20.72	3.82	14.61	38.45	0.05	-0.041	0.14	0.2837
Added SFA	117.87	24.43	61.76	173.25	0.006	-0.085	0.096	0.899
Added PUFA	90.02	44.34	14.56	192.87	0.287	0.201	0.368	<0.0001
Added TFA	7.24	3.2	2.18	20.14	-0.301	-0.381	-0.216	<0.0001
Alcohol	49.13	28.37	6.76	105.38	0.299	0.214	0.379	<0.0001
Sugary beverages	594.55	290.83	148.15	1183	0.133	0.043	0.221	0.0038
Potatoes	107.54	48.01	11.76	287.77	-0.073	-0.162	0.018	0.1168
Sweet potatoes	4.95	4.44	0.15	26.43	-0.041	-0.132	0.049	0.3715
Corn	58.76	48.17	13.34	228.27	-0.062	-0.151	0.029	0.1825
Fruits	64.36	16.74	23.05	161.39	-0.081	-0.17	0.01	0.0809
Vegetables	48.48	15.8	9.48	108.21	0.2	0.111	0.285	<0.0001
Nuts and seeds	2.92	4.64	0.26	28.08	0.237	0.15	0.321	<0.0001
Whole grains	58.04	18.5	14.07	89.85	0.023	-0.068	0.113	0.6258
Legumes	67.9	37.41	6.89	134.74	0.159	0.07	0.246	0.0005
Rice	75.96	35.41	14.07	159.66	-0.219	-0.304	-0.131	<0.0001
Animal food seven	354.17	81.09	176.03	567.08	0.526	0.457	0.588	<0.0001
Healthy plant seven	336.67	86.69	210.5	660.53	0.252	0.165	0.335	<0.0001
United Kingdom, n=66 cohorts	Mean	SD	Minimum	Maximum	r	95% CI low	95% CI high	P
NCD deaths/100000/year	1195	330.46	777.48	1823				
Processed meat	19.52	3.77	14.11	28.68	0.622	0.448	0.751	<0.0001
Red meat	95.95	21.67	66.41	138.19	0.78	0.663	0.86	<0.0001
Fish	10.59	1.92	7.84	15.96	0.264	0.023	0.476	0.0307
Milk	86.98	6.85	77.19	103.93	0.041	-0.203	0.28	0.7435
Poultry	71.9	16.9	53.59	113.58	-0.113	-0.345	0.133	0.366
Eggs	26.35	2.31	23.41	31.86	-0.131	-0.361	0.115	0.2924
Added SFA	157.55	4.65	150.15	166.99	0.24	-0.003	0.455	0.0507
Added PUFA	73.81	7.81	63.97	94.27	-0.013	-0.255	0.229	0.9152
Added TFA	9.53	1	8.61	13.08	-0.151	-0.379	0.095	0.2245
Alcohol	143.72	62.26	81.84	290.45	0.752	0.623	0.841	<0.0001
Sugary beverages	222.36	114.47	148.41	625.68	0.556	0.363	0.703	<0.0001
Potatoes	90.76	7.95	80.64	109.78	-0.131	-0.361	0.115	0.2926
Sweet potatoes	0.42	0.02	0.39	0.45	0.149	-0.097	0.377	0.2305
Corn	9.61	0.6	8.82	11.05	-0.134	-0.364	0.112	0.2819
Fruits	57.23	7.73	45.61	78.57	-0.563	-0.708	-0.372	<0.0001
Vegetables	92.89	9.52	80.5	117.82	0.12	-0.126	0.352	0.3348
Nuts and seeds	17.45	4.67	12.34	30.04	0.003	-0.239	0.245	0.9779
Whole grains	35.65	3.26	31.01	43.49	0.458	0.243	0.63	<0.0001
Legumes	20.04	2.16	14.93	24.03	0.758	0.632	0.845	<0.0001
Rice	14.2	0.35	13.72	15.01	-0.139	-0.369	0.107	0.2636
Animal food seven	468.82	48.3	401.45	599.19	0.391	0.164	0.578	0.001
Healthy plant seven	297.49	29.69	260.86	373.56	-0.006	-0.247	0.237	0.9646
United States, n=336 cohorts	Mean	SD	Minimum	Maximum	r	95% CI low	95% CI high	P
NCD deaths/100000/year	1197	322.46	696.87	2312				
Processed meat	39.9	12.28	22.25	68.77	0.811	0.771	0.845	<0.0001
Red meat	138.72	31.45	94.72	207.84	0.809	0.768	0.843	<0.0001
Alcohol	15.16	1.85	11.35	21.62	0.503	0.418	0.578	<0.0001
Milk	89.6	4.04	79.06	101.23	0.304	0.204	0.398	<0.0001
Poultry	190.9	0	190.9	190.9	NA	NA	NA	NA
Eggs	41.1	0	41.1	41.1	NA	NA	NA	NA
Added SFA	185.28	18.74	130.76	258.1	-0.143	-0.246	-0.036	0.0086
Added PUFA	164.94	10.7	116.52	186.09	0.025	-0.082	0.132	0.6492
Added TFA	25.52	3.18	16.44	37.19	-0.766	-0.807	-0.718	<0.0001
Alcohol	210.95	41.21	158.41	296.7	0.852	0.819	0.879	<0.0001
Sugary beverages	157.2	20.9	130.99	183.37	0.885	0.86	0.906	<0.0001
Potatoes	107.88	0	107.88	107.88	NA	NA	NA	NA
Sweet potatoes	3.49	0	3.49	3.49	NA	NA	NA	NA
Corn	23.94	0	23.94	23.94	NA	NA	NA	NA
Fruits	68.82	7.2	50.66	86.5	-0.581	-0.647	-0.505	<0.0001
Vegetables	114.74	30.87	64.95	224.91	-0.034	-0.14	0.074	0.5385
Nuts and seeds	25.66	3.58	17.87	38.04	0.158	0.052	0.261	0.0036
Whole grains	54.81	2.35	48.91	61.15	0.521	0.438	0.595	<0.0001
Legumes	35.82	3.12	31.44	39.69	0.884	0.858	0.905	<0.0001
Rice	17.29	0	17.29	17.29	NA	NA	NA	NA
Animal food seven	700.66	47.69	586.76	790.13	0.731	0.677	0.777	<0.0001
Healthy plant seven	468.28	40.49	382.87	604.94	-0.01	-0.117	0.097	0.85
Mexico, n=130 cohorts	Mean	SD	Minimum	Maximum	r	95% CI low	95% CI high	P
NCD deaths/100000/year	1101	211.47	687.38	1557				
Processed meat	11.55	3.93	3.98	26.52	-0.025	-0.196	0.148	0.7774
Red meat	64.65	14.01	41.88	113.15	0.81	0.741	0.862	<0.0001
Fish	8.96	1.63	5.89	11.95	0.694	0.592	0.774	<0.0001
Milk	34.93	3.15	27.32	42.97	0.423	0.271	0.555	<0.0001
Poultry	76.14	19.03	41.48	110.41	0.417	0.264	0.55	<0.0001
Eggs	34.63	3.61	26.96	40.6	0.416	0.262	0.549	<0.0001
Added SFA	107.49	5.83	89.06	114.89	0.284	0.118	0.435	0.0009
Added PUFA	63.99	8.52	49.45	77.73	0.462	0.315	0.588	<0.0001
Added TFA	35.02	4.51	25.63	42.56	-0.394	-0.53	-0.238	<0.0001
Alcohol	62.21	49.4	4.25	222.94	0.548	0.415	0.658	<0.0001
Sugary beverages	892.95	205.34	401.93	1472	0.164	-0.008	0.327	0.0605
Potatoes	22.38	2.37	17.26	26.3	0.415	0.261	0.548	<0.0001
Sweet potatoes	0.59	0.03	0.54	0.66	-0.41	-0.544	-0.256	<0.0001
Corn	209.53	16.73	171.66	236.88	0.413	0.259	0.546	<0.000
Fruits	66.77	9.58	47.43	85.51	0.005	-0.167	0.177	0.9516
Vegetables	62.15	7.17	49.16	80.7	0.444	0.294	0.572	<0.0001
Nuts and seeds	6.59	1.89	3.34	14.02	0.499	0.358	0.618	<0.0001
Whole grains	144.18	17.46	109.99	235.1	0.589	0.464	0.691	<0.0001
Legumes	77.19	6.43	69.15	86.31	0.799	0.727	0.854	<0.0001
Rice	12.09	0.38	11.2	12.69	0.414	0.26	0.547	<0.0001
Animal food seven	338.35	41	254.67	416.12	0.605	0.483	0.704	<0.0001
Healthy plant seven	421.46	36.3	340.61	506.87	0.648	0.536	0.738	<0.0001
Japan, n=158 cohorts	Mean	SD	Minimum	Maximum	r	95% CI low	95% CI high	P
NCD deaths/100000/year	725.61	263.67	423.8	1186				
Processed meat	19.19	3	14.16	27.62	0.633	0.529	0.718	<0.0001
Red meat	58.72	12.94	41.43	85.87	0.896	0.86	0.923	<0.0001
Meat (processed+red meat)	77.92	15.67	55.66	113.5	0.861	0.815	0.897	<0.0001
Fish	260.54	36.18	195.66	370.36	0.613	0.505	0.702	<0.0001
Milk	29.04	2.33	25.35	35.4	-0.384	-0.509	-0.242	<0.0001
Poultry	63.19	14.2	45.68	104.37	-0.017	-0.172	0.14	0.8367
Eggs	51.87	4.12	45.93	63.43	-0.017	-0.173	0.14	0.833
Added SFA	93.95	3.61	71.94	103.96	-0.145	-0.295	0.011	0.068
Added PUFA	65	6.72	54.33	84.79	0.085	-0.073	0.238	0.2898
Added TFA	4.27	0.45	3.61	5.15	-0.916	-0.938	-0.886	<0.0001
Alcohol	183.16	13.98	164.35	206.01	0.961	0.947	0.971	<0.0001
Sugary beverages	94.78	20.62	72.91	117.58	0.961	0.947	0.971	<0.0001
Potatoes	42	3.35	37.18	51.38	-0.017	-0.173	0.14	0.833
Sweet potatoes	12.06	0.4	10.98	12.72	0.017	-0.14	0.173	0.8328
Corn	27.42	1.58	25.08	31.81	-0.017	-0.173	0.14	0.8327
Fruits	44.43	7.45	31.67	63.58	-0.738	-0.802	-0.658	<0.0001
Vegetables	149.32	13.07	125.6	188.72	0.25	0.097	0.391	0.0014
Nuts and seeds	9.26	2.34	5.47	16.19	-0.002	-0.159	0.154	0.9752
Whole grains	76.05	4.64	67.16	89.63	0.507	0.381	0.614	<0.0001
Legumes	71.84	3.96	65.43	79.06	0.942	0.921	0.957	<0.0001
Rice	123.91	2.75	119.66	131.48	-0.017	-0.173	0.14	0.8325
Animal food seven	576.5	67.57	464.86	786.29	0.502	0.375	0.61	<0.0001
Healthy plant seven	427.97	30.01	376.23	515.56	0.147	-0.009	0.296	0.0642
SDI top quartile, n=1926 cohorts	Mean	SD	Minimum	Maximum	r	95% CI low	95% CI high	P
NCD deaths/100000/year	1288	607.73	423.8	3349				
Processed meat	16.59	14.56	0.49	68.77	-0.036	-0.08	0.009	0.115
Red meat	96.29	47.64	7.62	235.95	0.149	0.105	0.193	<0.0001
Fish	30.76	69.59	0.87	370.36	-0.265	-0.306	-0.223	<0.0001
Milk	61.29	30.13	1.44	146.82	-0.117	-0.161	-0.073	<0.0001
Poultry	82.11	42.89	7.53	289.96	-0.156	-0.199	-0.112	<0.0001
Eggs	29.31	9.98	2.34	63.43	-0.141	-0.184	-0.096	<0.0001
Added SFA	143.07	43.88	30.65	316.82	-0.139	-0.183	-0.095	<0.0001
Added PUFA	90.63	47.61	1.45	212.01	-0.238	-0.28	-0.195	<0.0001
Added TFA	12.14	10.21	1.33	42.56	-0.21	-0.252	-0.166	<0.0001
Alcohol	116.43	74.52	6.67	429.81	0.051	0.006	0.095	0.0255
Sugary beverages	297.65	204.49	72.91	1468	0.229	0.186	0.271	<0.0001
Potatoes	95.39	55.51	4.04	286.52	0.447	0.411	0.482	<0.0001
Sweet potatoes	3.33	6.43	0.02	98.95	-0.141	-0.185	-0.097	<0.0001
Corn	28.73	54.08	0.16	343.7	-0.025	-0.069	0.02	0.2823
Fruits	60.76	20.31	7.23	160.35	-0.471	-0.505	-0.435	<0.0001
Vegetables	119.59	45.69	16.31	304.17	-0.033	-0.078	0.011	0.1446
Nuts and seeds	17.3	11.43	0.05	102.99	-0.258	-0.299	-0.216	<0.0001
Whole grains	46.72	33.77	1.55	235.1	-0.182	-0.225	-0.138	<0.0001
Legumes	39.07	26.84	0.64	133.5	-0.183	-0.226	-0.139	<0.0001
Rice	36.63	50.55	3.14	237.22	-0.215	-0.257	-0.172	<0.0001
Animal food seven	459.42	150.27	54.03	786.29	-0.197	-0.239	-0.154	<0.0001
Healthy plant seven	377.41	104.63	138.4	737.86	-0.357	-0.395	-0.317	<0.0001
SDI bottom three quartiles, n=5920 cohorts	Mean	SD	Minimum	Maximum	r	95% CI low	95% CI high	P
NCD deaths/100000/year	1474	394.3	625.82	4321				
Processed meat	1.67	1.29	0.2	26.52	-0.185	-0.209	-0.16	<0.0001
Red meat	35.3	32.38	3.21	192.51	-0.148	-0.172	-0.123	<0.0001
Fish	3.23	2.51	0.4	27.73	-0.209	-0.233	-0.184	<0.0001
Milk	13.24	10.4	1.06	103.76	0.048	0.023	0.073	0.0002
Poultry	26.22	27.24	1.4	219.04	-0.306	-0.329	-0.283	<0.0001
Eggs	14.8	12.05	1.05	38.45	-0.37	-0.392	-0.348	<0.0001
Added SFA	93.61	29.63	18.28	314.76	-0.13	-0.155	-0.105	<0.0001
Added PUFA	29.3	25.74	1.63	192.87	-0.212	-0.236	-0.187	<0.0001
Added TFA	6.01	5.73	0.69	39.35	0.038	0.012	0.063	0.0038
Alcohol	69.52	44.82	4.25	340.37	0.335	0.312	0.358	<0.0001
Sugary beverages	298.59	131.05	171.24	1472	0.02	-0.006	0.045	0.1263
Potatoes	80.9	88.83	3.49	666.7	-0.053	-0.079	-0.028	<0.0001
Sweet potatoes	31.52	42.01	0.04	438.25	-0.188	-0.213	-0.164	<0.0001
Corn	38.7	50.45	0.29	351.5	-0.068	-0.094	-0.043	<0.0001
Fruits	33.52	18.84	3.58	161.39	-0.367	-0.389	-0.345	<0.0001
Vegetables	66.8	33.18	9.48	198.51	-0.078	-0.104	-0.053	<0.0001
Nuts and seeds	5.52	4	0.05	49.94	-0.044	-0.07	-0.019	0.0006
Whole grains	58.55	29.37	1.14	165.51	-0.151	-0.176	-0.126	<0.0001
Legumes	55.86	32.75	0.51	194.7	0.164	0.14	0.189	<0.0001
Rice	189.53	126.84	2.33	547.15	-0.012	-0.038	0.013	0.3377
Animal food seven	188.07	73.08	60.63	567.08	-0.297	-0.32	-0.273	<0.0001
Healthy plant seven	281.07	73.66	63.27	660.53	-0.3	-0.323	-0.277	<0.0001

Multiple regression-derived formulas of risk factors versus NCDs

With methods detailed in Appendices, we derived a multiple regression risk factor formula with risk factors from the lowest 416 NCD cohort pairs with mean m/f animal food seven of <400 KC/d (Table 7) together with m/f pairs of cohorts with mean animal food seven of <149 KC/d (the lowest mean m/f animal food seven {KC/d} in Table 3, Kenya). Table 11 gives the basic statistics for this subset (n=2724 cohorts, representing about 2.7 billion people).

**Table 11:** GBD subset for multiple regression formula derivation with low animal food seven ^a^See Appendices for the methodology of deriving multiple regression risk factor formulas NCD, noncommunicable disease; SD, standard deviation; SFA, saturated fatty acid; PUFA, polyunsaturated fatty acid; TFA, trans fatty acid; METs, metabolic equivalent of tasks; PM_0.25_, particulate matter 0.25; BMI, body mass index; LDLc, low-density lipoprotein cholesterol; FPG, fasting plasma glucose; SBP, systolic blood pressure; KC/d, kilocalories/day; GBD, Global Burden of Disease

NCD deaths/100000/year versus risk factors, NCDs of <1070.22659 deaths/100000/year or animal food seven of <149 KC/d, n=2724 cohorts, and all variables as means males/females^a^	Mean	SD	Minimum	Maximum	r	95% CI low	95% CI high	P
NCD deaths/100000/year	1545	343.58	713.37	3521				
Processed meat, KC/d	1.46	1.35	0.28	13.26	-0.399	-0.430	-0.367	<0.0001
Red meat, KC/d	17.01	17.83	4.85	108.84	-0.445	-0.474	-0.414	<0.0001
Fish, KC/d	2.52	2.24	0.44	11.52	-0.610	-0.633	-0.586	<0.0001
Milk, KC/d	15.98	8.87	1.08	76.05	-0.427	-0.458	-0.396	<0.0001
Poultry, KC/d	17.53	26.82	1.40	143.64	-0.614	-0.637	-0.590	<0.0001
Eggs, KC/d	7.07	6.08	1.05	38.45	-0.643	-0.664	-0.620	<0.0001
Added SFA, KC/d	85.54	24.23	18.30	173.02	-0.465	-0.494	-0.435	<0.0001
Added PUFA, KC/d	27.62	23.77	1.65	142.96	-0.627	-0.650	-0.604	<0.0001
Added TFA, KC/d	9.15	7.20	0.77	36.96	-0.150	-0.187	-0.113	<0.0001
Alcohol, KC/d	77.05	34.76	9.53	246.48	0.132	0.095	0.169	<0.0001
Sugary beverages, KC/d	315.61	144.71	98.34	1392	-0.431	-0.461	-0.400	<0.0001
Potatoes, KC/d	83.22	101.35	3.49	606.10	-0.103	-0.140	-0.065	<0.0001
Sweet potatoes, KC/d	14.33	31.03	0.16	226.79	0.052	0.014	0.089	0.007
Corn, KC/d	45.13	63.51	0.54	351.50	-0.092	-0.129	-0.055	<0.0001
Fruits, KC/d	32.97	22.49	3.84	110.73	-0.514	-0.541	-0.486	<0.0001
Vegetables, KC/d	64.20	40.64	9.62	192.32	-0.199	-0.235	-0.163	<0.0001
Nuts/seeds, KC/d	5.77	4.67	0.27	31.59	-0.176	-0.213	-0.140	<0.0001
Whole grains, KC/d	59.54	28.70	2.30	150.57	-0.148	-0.184	-0.111	<0.0001
Legumes, KC/d	74.47	31.38	11.52	180.77	0.025	-0.013	0.062	0.2000
Rice, KC/d	180.24	139.66	2.33	547.15	0.131	0.094	0.168	<0.0001
Animal food seven, KC/d	147.09	67.88	56.29	396.61	-0.667	-0.687	-0.645	<0.0001
Healthy plant seven, KC/d	278.90	78.63	84.22	595.12	-0.474	-0.502	-0.444	<0.0001
Total KC/d	2345	337	1579	3254	-0.503	-0.531	-0.474	<0.0001
Vitamin A deficiency in children/100000/year	29869	9317	1475	48691	0.449	0.418	0.478	<0.0001
Sodium, gram/day	3.09	0.67	1.57	6.42	-0.108	-0.145	-0.071	<0.0001
Calcium, gram/day	0.219	0.072	0.087	0.725	-0.536	-0.563	-0.509	<0.0001
Fiber, gram/day	9.41	3.22	2.94	20.95	-0.159	-0.195	-0.122	<0.0001
Physical activity METs	4639	1119	2144	7496	0.185	0.148	0.221	<0.0001
Child underweight of >2 SD	0.346	0.164	0.014	0.497	0.493	0.464	0.521	<0.0001
Stop breast feeding in <6 months	0.077	0.039	0.016	0.226	-0.575	-0.600	-0.549	<0.0001
Ambient pollution, PM_0.25_	62.12	26.19	8.87	95.54	0.322	0.288	0.355	<0.0001
Smoking rate (0-1)	0.148	0.051	0.042	0.314	-0.097	-0.134	-0.060	<0.0001
Secondhand smoke (0-1)	0.361	0.075	0.178	0.497	-0.020	-0.058	0.017	0.2939
Sublingual tobacco (0-1)	0.145	0.111	0.002	0.297	0.280	0.245	0.315	<0.0001
Blood lead, mcg/dL	5.29	0.65	3.72	7.35	0.130	0.093	0.167	<0.0001
Household air pollution (0-1)	0.669	0.270	0.006	0.996	0.522	0.494	0.548	<0.0001
Kidney disease III (0-1)	0.071	0.014	0.031	0.108	0.094	0.057	0.131	<0.0001
BMI kg/m^2^	20.22	1.94	18.28	25.98	-0.553	-0.579	-0.527	<0.0001
LDLc, mmol/L	4.13	0.26	3.40	4.99	-0.356	-0.388	-0.323	<0.0001
FPG, mmol/L	2.05	0.27	1.34	3.02	-0.525	-0.551	-0.497	<0.0001
SBP, mm Hg	132.98	3.26	124.71	144.28	-0.052	-0.089	-0.014	0.0066
Sociodemographic index (0-1)	0.411	0.113	0.112	0.792	-0.626	-0.648	-0.602	<0.0001

Table 12 shows the three-step derivation of the multiple regression risk factor formula from this low animal food seven subset with paired risk factors.

**Table 12:** Multiple regression formula derivation 1: low animal food seven, m/f paired ^d^See Appendices for the methodology of deriving multiple regression risk factor formulas ^e^Three confounded dietary variables received partial R-squared values instead of parameter estimates in the preliminary risk factor formula KC/d, kilocalories/day; SFA, saturated fatty acid; PUFA, polyunsaturated fatty acid; TFA, trans fatty acid; SD, standard deviation; PM_0.25_, particulate matter 0.25; PAR%, population-attributable risk percent; m/f, male/female; SAS, Statistical Analysis System

Step 1^d^																						
Combination risk factors			Standardized relevant variables												Mean					R^2^		
Diet 1	-		Processed meat, KC/d										*		1.4574		*			0.1593		
Diet 1	-		Red meat, KC/d										*		17.0053		*			0.1977		
Diet 1	-		Fish, KC/d										*		2.5162		*			0.3721		
Diet 1	-		Milk, KC/d										*		15.9755		*			0.1826		
Diet 1	-		Poultry, KC/d										*		17.5265		*			0.3769		
Diet 1	-		Eggs, KC/d										*		7.0705		*			0.4134		
Diet 1	-		Added SFA, KC/d										*		85.5365		*			0.2163		
Diet 1	-		Added PUFA, KC/d										*		27.6228		*			0.3936		
Diet 1	-		Added TFA, KC/d										*		9.1538		*			0.0225		
Diet 1	+		Alcohol, KC/d										*		77.0467		*			0.0175		
Diet 1	-		Sugary beverages, KC/d										*		315.6125		*			0.1858		
Diet 1	-		Potatoes, KC/d										*		83.2168		*			0.0106		
Diet 1	+		Sweet potatoes, KC/d										*		14.328		*			0.0027		
Diet 1	-		Corn, KC/d										*		45.1333		*			0.0085		
Diet 1	-		Fruits, KC/d										*		32.966		*			0.2641		
Diet 1	-		Vegetables, KC/d										*		64.2021		*			0.0397		
Diet 1	-		Nuts/seeds, KC/d										*		5.7717		*			0.0311		
Diet 1	-		Whole grains, KC/d										*		59.5355		*			0.0219		
Diet 1	+		Legumes, KC/d										*		74.4707		*			0.0006		
Diet 1	+		Rice, KC/d										*		180.2407		*			0.0173		
Nondiet 1	+		Vitamin A deficiency in age of <5 years										*		1		*			0.2014		
Nondiet 1	+		Child underweight of >2 SD										*		0.3463		*			0.2431		
Nondiet 1	+		Ambient pollution PM_0.25_										*		1		*			0.1035		
Nondiet 1	+		Sublingual tobacco (0-1)										*		0.1451		*			0.0786		
Nondiet 1	+		Blood lead, mcg/dL										*		1		*			0.0169		
Nondiet 1	+		Household air pollution (0-1)										*		0.6689		*			0.2721		
Nondiet 1	+		Kidney disease III (0-1)										*		0.0709		*			0.0089		
Nondiet 1	+		Sociodemographic index (0-1)										*		0.411		*			0.3915		
Step 2																						
Combination risk factors and individual dietary risk factors				Standardized variables					Mean			R^2^				Multiple regression parameter estimates and partial R^2^ per SAS					Preliminary risk factor formula 1	
Diet 2		-		Processed meat, KC/d			*		1.45735		*	0.1593		*		0.0114		=			0.00265	
Diet 2		-		Red meat, KC/d			*		17.0053		*	0.1977		*		0.0114		=			0.03833	
Diet 2		-		Fish, KC/d			*		2.51617		*	0.3721		*		0.0114		=			0.01067	
Diet 2		-		Milk, KC/d			*		15.97554		*	0.1826		*		0.0114		=			0.03326	
Diet 2		-		Poultry, KC/d			*		17.52645		*	0.3769		*		0.0114		=			0.0753	
Diet 2		-		Eggs, KC/d			*		7.07047		*	0.4134		*		0.0114		=			0.03332	
Diet 2		-		Added SFA, KC/d			*		85.53648		*	0.2163		*		0.0114		=			0.2109	
Diet 2		-		Added PUFA, KC/d			*		27.62283		*	0.3936		*		0.0114		=			0.12394	
Diet 2		-		Added TFA, KC/d			*		9.15384		*	0.0225		*		0.0114		=			0.00235	
Diet 2		+		Alcohol, KC/d			*		77.04666		*	0.0175		*		0.0114		=			0.01536	
Sugary beverages^e^		+		Sugary beverages, KC/d^e^			*		315.6125		*	0.1858		*		0.0019		=			0.0019	
Diet 2		-		Potatoes, KC/d			*		83.21676		*	0.0106		*		0.0114		=			0.01001	
Sweet potatoes^e^		-		Sweet potatoes, KC/d^e^			*		14.32801		*	0.0027		*		0		=			0	
Diet 2		-		Corn, KC/d			*		45.13329		*	0.0085		*		0.0114		=			0.00437	
Diet 2		-		Fruits, KC/d			*		32.96595		*	0.2641		*		0.0114		=			0.09927	
Diet 2		-		Vegetables, KC/d			*		64.20206		*	0.0397		*		0.0114		=			0.02903	
Diet 2		-		Nuts/seeds, KC/d			*		5.77165		*	0.0311		*		0.0114		=			0.00205	
Diet 2		-		Whole grains, KC/d			*		59.53552		*	0.0219		*		0.0114		=			0.01483	
Legumes^e^		-		Legumes, KC/d^e^			*		74.47066		*	0.0006		*		0.0047		=			0.0047	
Rice		+		Rice, KC/d			*		180.2407		*	0.0173		*		0.0114		=			0.03544	
Nondiet 2		+		Vitamin A deficiency in age of <5 years			*		1		*	0.2014		*		0.57013		=			0.11482	
Nondiet 2		+		Child underweight of >2 SD			*		0.34628		*	0.2431		*		0.57013		=			0.048	
Nondiet 2		+		Ambient pollution PM_0.25_			*		1		*	0.1035		*		0.57013		=			0.059	
Nondiet 2		+		Sublingual tobacco (0-1)			*		0.14512		*	0.0786		*		0.57013		=			0.0065	
Nondiet 2		+		Blood lead, mcg/dL			*		1		*	0.0169		*		0.57013		=			0.00962	
Nondiet 2		+		Household air pollution (0-1)			*		0.66893		*	0.2721		*		0.57013		=			0.10378	
Nondiet 2		+		Kidney disease III (0-1)			*		0.07091		*	0.0089		*		0.57013		=			0.00036	
Nondiet 2		-		Sociodemographic index (0-1)			*		0.41101		*	0.3915		*		0.57013		=			0.09174	
Step 3																						
Standardized variables						Preliminary risk factor formula				Standardized variables												Final risk factor formula
Processed meat, KC/d					*	0.002646		-		Processed meat, KC/d									*			0.12
Red meat, KC/d					*	0.038325		-		Red meat, KC/d									*			1.7
Fish, KC/d					*	0.010672		-		Fish, KC/d									*			0.47
Milk, KC/d					*	0.033257		-		Milk, KC/d									*			1.48
Poultry, KC/d					*	0.075305		-		Poultry, KC/d									*			3.35
Eggs, KC/d					*	0.033318		-		Eggs, KC/d									*			1.48
Added SFA, KC/d					*	0.210899		-		Added SFA, KC/d									*			9.38
Added PUFA, KC/d					*	0.123943		-		Added PUFA, KC/d									*			5.51
Added TFA, KC/d					*	0.00235		-		Added TFA, KC/d									*			0.1
Alcohol, KC/d					*	0.015362		+		Alcohol, KC/d									*			0.68
Sugary beverages, KC/d					*	0.0019		+		Sugary beverages, KC/d									*			0.08
Potatoes, KC/d					*	0.010012		-		Potatoes, KC/d									*			0.45
Sweet potatoes, KC/d					*	0		-		Sweet potatoes, KC/d									*			0
Corn, KC/d					*	0.004369		-		Corn, KC/d									*			0.19
Fruits, KC/d					*	0.099269		-		Fruits, KC/d									*			4.41
Vegetables, KC/d					*	0.029034		-		Vegetables, KC/d									*			1.29
Nuts/seeds, KC/d					*	0.002047		-		Nuts/seeds, KC/d									*			0.09
Whole grains, KC/d					*	0.014834		-		Whole grains, KC/d									*			0.66
Legumes, KC/d					*	0.0047		-		Legumes, KC/d									*			0.21
Rice, KC/d					*	0.035445		+		Rice, KC/d									*			1.58
Vitamin A deficiency in age of <5 years					*	0.114821		+		Vitamin A deficiency in age of <5 years									*			5.11
Child underweight of >2 SD					*	0.048001		+		Child underweight of >2 SD									*			2.13
Ambient pollution, PM_0.25_					*	0.059003		+		Ambient pollution, PM_0.25_									*			2.62
Sublingual tobacco (0-1)					*	0.006501		+		Sublingual tobacco (0-1)									*			0.29
Blood lead mcg/dL					*	0.009616		+		Blood lead, mcg/dL									*			0.43
Household air pollution (0-1)					*	0.103784		+		Household air pollution (0-1)									*			4.61
Kidney disease III (0-1)					*	0.00036		+		Kidney disease III (0-1)									*			0.02
Sociodemographic index (0-1)					*	0.091737		-		Sociodemographic index (0-1)									*			4.08
Sum						1.18151				Total formula PAR%												52.53
Sum						1.18151																
r						0.72481																
R^2^						0.52535																
R^2^/sum						0.44464																
All risk factors (three)=all risk factors (two)*R^2^/sum*100						44.46425																

The resulting multiple regression formula is as follows: NCD risk factor formula for low animal food seven cohorts=-0.12*processed meat-1.70*red meat-0.47*Fish-1.48*milk-3.35*poultry-1.48*eggs-9.38*added SFA-5.51*added PUFA-0.10*added TFA+0.68*alcohol+0.08*sugary beverages-0.45*potatoes-0.00*sweet potatoes-0.19*corn-4.41*fruit-1.29*vegetables-0.09*nuts and seeds-0.66*whole grains-0.21*legumes+1.58*rice+5.11*vitamin A deficiency in children+2.13*severe underweight children+2.62*ambient air pollution+0.29*sublingual tobacco use+0.43*blood lead level+4.61*household air pollution+0.02*kidney disease stage III-4.08*sociodemographic index. The total PAR% of risk factors=52.53%; mean NCD=1545 deaths/100000/year.

Table 13 shows the same subset as Table 12 but with male and female cohorts unpaired.

**Table 13:** Low animal food seven subset basic statistics with male and female cohorts unpaired NCD, noncommunicable disease; SD, standard deviation; CI, confidence interval; KC/d, kilocalories/day; SFA, saturated fatty acid; PUFA, polyunsaturated fatty acid; TFA, trans fatty acid; METs, metabolic equivalent of tasks; PM_0.25_, particulate matter 0.25; BMI, body mass index; LDL, low-density lipoprotein; BP, blood pressure

NCD deaths/100000/year versus risk factors, NCDs of <1070.22659 or animal food seven of <149 KC/d, n=2724 cohorts	Mean	SD	Minimum	Maximum	r	95% CI low	95% CI high	P
NCD deaths/100000/year	1545	415.22	582.18	4321				
Processed meat, KC/d	1.45	1.38	0.24	17.49	-0.269	-0.304	-0.234	<0.0001
Red meat, KC/d	17.00	18.57	3.84	130.56	-0.268	-0.302	-0.232	<0.0001
Fish, KC/d	2.51	2.25	0.40	12.48	-0.461	-0.490	-0.431	<0.0001
Milk, KC/d	15.97	8.88	1.06	78.07	-0.335	-0.368	-0.301	<0.0001
Poultry, KC/d	17.53	26.82	1.40	143.64	-0.508	-0.535	-0.480	<0.0001
Eggs, KC/d	7.07	6.08	1.05	38.45	-0.532	-0.558	-0.505	<0.0001
Added SFA, KC/d	85.53	25.33	16.78	187.30	-0.245	-0.280	-0.209	<0.0001
Added PUFA, KC/d	27.65	24.05	1.49	156.98	-0.465	-0.494	-0.435	<0.0001
Added TFA, KC/d	9.07	7.14	0.75	37.50	-0.136	-0.173	-0.099	<0.0001
Alcohol, KC/d	77.03	44.13	4.25	316.45	0.332	0.299	0.365	<0.0001
Sugary beverages, KC/d	315.60	154.45	76.43	1472	-0.191	-0.227	-0.155	<0.0001
Potatoes, KC/d	83.22	101.35	3.49	606.10	-0.085	-0.122	-0.048	<0.0001
Sweet potatoes, KC/d	14.33	31.03	0.16	226.79	0.043	0.005	0.080	0.0256
Corn, KC/d	45.13	63.51	0.54	351.50	-0.076	-0.113	-0.039	<0.0001
Fruits, KC/d	32.97	22.64	3.58	117.72	-0.462	-0.491	-0.432	<0.0001
Vegetables, KC/d	64.20	40.74	9.48	198.51	-0.134	-0.171	-0.097	<0.0001
Nuts and seeds, KC/d	5.77	4.68	0.26	32.59	-0.130	-0.167	-0.093	<0.0001
Whole grains, KC/d	59.54	28.85	2.22	165.14	-0.085	-0.122	-0.048	<0.0001
Legumes, KC/d	74.47	32.38	10.67	194.70	0.123	0.086	0.159	<0.0001
Rice, KC/d	180.24	139.66	2.33	547.15	0.109	0.071	0.146	<0.0001
Animal food seven, KC/d	147.09	67.88	56.29	396.61	-0.552	-0.577	-0.525	<0.0001
Healthy plants, KC/d	278.90	78.63	84.22	595.12	-0.392	-0.423	-0.360	<0.0001
Total KC/d available	2345	337	1579	3254	-0.416	-0.447	-0.385	<0.0001
Vitamin A deficiency in age of <5	29869	9340	1368	48763	0.390	0.357	0.421	<0.0001
Sodium, gram/day	3.09	0.69	1.33	6.54	-0.084	-0.121	-0.046	<0.0001
Calcium, gram/day	0.22	0.07	0.08	0.76	-0.371	-0.403	-0.338	<0.0001
Dietary fiber, gram/day	9.41	3.33	2.72	22.68	-0.028	-0.065	0.010	0.1471
Physical activity METs	4639	1319	1652	7607	0.312	0.278	0.345	<0.0001
Child underweight of >2 SD	0.343	0.162	0.013	0.488	0.422	0.391	0.452	<0.0001
Stop breast feeding in <6 months	0.074	0.040	0.016	0.226	-0.477	-0.506	-0.448	<0.0001
Ambient pollution, PM_0.25_	62.12	26.19	8.87	95.54	0.274	0.239	0.309	<0.0001
Smoking rate (0-1)	0.145	0.135	0.003	0.489	0.432	0.401	0.462	<0.0001
Secondhand smoking (0-1)	0.358	0.126	0.164	0.709	-0.422	-0.452	-0.391	<0.0001
Sublingual tobacco rate (0-1)	0.142	0.121	0.001	0.419	0.361	0.327	0.393	<0.0001
Blood lead level, mcg/dL	5.29	0.77	3.38	8.37	0.304	0.270	0.338	<0.0001
Household air pollution (0-1)	0.666	0.270	0.006	0.996	0.428	0.397	0.458	<0.0001
Kidney disease stage III (0-1)	0.068	0.020	0.025	0.134	-0.243	-0.278	-0.207	<0.0001
BMI, kg/m^2^	20.22	1.99	17.95	26.53	-0.530	-0.556	-0.502	<0.0001
LDL cholesterol, mmol/L	2.05	0.28	1.27	3.05	-0.516	-0.543	-0.488	<0.0001
Fasting plasma glucose, mmol/L	4.13	0.26	3.36	5.06	-0.244	-0.279	-0.209	<0.0001
Systolic BP, mm Hg	132.98	3.53	123.41	145.74	-0.133	-0.170	-0.096	<0.0001
Sociodemographic index (0-1)	0.411	0.113	0.112	0.792	-0.518	-0.545	-0.490	<0.0001
Sex: male, one; female, two	1.500	0.500	1.000	2.000	-0.458	-0.488	-0.428	<0.0001

Table 14 shows the derivation for the above subset (n=2724 cohorts) with individual cohorts.

**Table 14:** Multiple regression formula derivation 2: low animal food seven, m/f unpaired ^d^See Appendices for the methodology of deriving multiple regression risk factor formulas ^e^Three confounded dietary variables received partial R-squared values instead of parameter estimates in the preliminary risk factor formula KC/d, kilocalories/day; SFA, saturated fatty acid; PUFA, polyunsaturated fatty acid; TFA, trans fatty acid; SD, standard deviation; PM_0.25,_ particulate matter 0.25; PAR%, population-attributable risk percent; m/f, male/female

Step 1^d^																							
Combination risk factors					Standardized variables							Mean							R^2^				
Diet 1				-	Processed meat, KC/d					*		1.45412						*	0.07254				
Diet 1				-	Red meat, KC/d					*		17.00207						*	0.07166				
Diet 1				-	Fish, KC/d					*		2.51294						*	0.21233				
Diet 1				-	Milk, KC/d					*		15.97231						*	0.11215				
Diet 1				-	Poultry, KC/d					*		17.52645						*	0.25812				
Diet 1				-	Eggs, KC/d					*		7.07047						*	0.28309				
Diet 1				-	Added SFA, KC/d					*		85.52853						*	0.05991				
Diet 1				-	Added PUFA, KC/d					*		27.65038						*	0.21648				
Diet 1				-	Added TFA, KC/d					*		9.07367						*	0.01852				
Diet 1				+	Alcohol, KC/d					*		77.02586						*	0.11058				
Diet 1				-	Sugary beverages, KC/d^e^					*		315.60059						*	0.03649				
Diet 1				-	Potatoes, KC/d					*		83.21676						*	0.00723				
Diet 1				+	Sweet potatoes, KC/d^e^					*		14.32801						*	0.00183				
Diet 1				-	Corn, KC/d					*		45.13329						*	0.00582				
Diet 1				-	Fruits, KC/d					*		32.96595						*	0.21387				
Diet 1				-	Vegetables, KC/d					*		64.20206						*	0.01799				
Diet 1				-	Nuts and seeds, KC/d					*		5.77165						*	0.01688				
Diet 1				-	Whole grains, KC/d					*		59.53552						*	0.00728				
Diet 1				+	Legumes, KC/d^e^					*		74.47066						*	0.01506				
Diet 1				+	Rice, KC/d					*		180.24074						*	0.01182				
Nondiet 1				+	Vitamin A deficiency in age of <5 years					*		0.2987						*	0.1519				
Nondiet 1				+	Child underweight of >2 SD					*		0.3433						*	0.1780				
Nondiet 1				+	Ambient pollution, PM_0.25_					*		1.0000						*	0.0752				
Nondiet 1				+	Smoking rate (0-1)					*		0.1451						*	0.1866				
Nondiet 1				+	Sublingual tobacco rate (0-1)					*		0.1422						*	0.1300				
Nondiet 1				+	Blood lead level, mcg/dL					*		1.0000						*	0.0927				
Nondiet 1				+	Household air pollution (0-1)					*		0.6660						*	0.1829				
Nondiet 1				-	Sociodemographic index (0-1)					*		0.4110						*	0.2680				
Sex (m/f)				-	Sex: male, one; female, two					*		1.0000						*	0.2101				
Step 2																							
Dietary risk factor combination variable and independent dietary risk factors			Standardized variables						Mean						R^2^		Multiple regression parameter estimates and partial R^2^					Preliminary risk factor formula	
Diet 2		-	Processed meat, KC/d					*	1.4541				*		0.0725	*	0.01165			=		0.0012	
Diet 2		-	Red meat, KC/d					*	17.0021				*		0.0717	*	0.01165			=		0.0142	
Diet 2		-	Fish, KC/d					*	2.5129				*		0.2123	*	0.01165			=		0.0062	
Diet 2		-	Milk, KC/d					*	15.9723				*		0.1122	*	0.01165			=		0.0209	
Diet 2		-	Poultry, KC/d					*	17.5265				*		0.2581	*	0.01165			=		0.0527	
Diet 2		-	Eggs, KC/d					*	7.0705				*		0.2831	*	0.01165			=		0.0233	
Diet 2		-	Added SFA, KC/d					*	85.5285				*		0.0599	*	0.01165			=		0.0597	
Diet 2		-	Added PUFA, KC/d					*	27.6504				*		0.2165	*	0.01165			=		0.0697	
Diet 2		-	Added TFA, KC/d					*	9.0737				*		0.0185	*	0.01165			=		0.0020	
Diet 2		+	Alcohol, KC/d					*	77.0259				*		0.1106	*	0.01165			=		0.0992	
Sugary beverages^e^		+	Sugary beverages, KC/d^e^					*	315.6006				*		0.0365	*	0.01165			=		0.0000	
Diet 2		-	Potatoes, KC/d					*	83.2168				*		0.0072	*	0.01165			=		0.0070	
Sweet potatoes^e^		-	Sweet potatoes, KC/d^e^					*	14.3280				*		0.0018	*	0.01165			=		0.0041	
Diet 2		-	Corn, KC/d					*	45.1333				*		0.0058	*	0.01165			=		0.0031	
Diet 2		-	Fruits, KC/d					*	32.9660				*		0.2139	*	0.01165			=		0.0821	
Diet 2		-	Vegetables, KC/d					*	64.2021				*		0.0180	*	0.01165			=		0.0135	
Diet 2		-	Nuts and seeds, KC/d					*	5.7717				*		0.0169	*	0.01165			=		0.0011	
Diet 2		-	Whole grains, KC/d					*	59.5355				*		0.0073	*	0.01165			=		0.0050	
Legumes ^e^		-	Legumes, KC/d^e^					*	74.4707				*		0.0151	*	0.01165			=		0.0149	
Diet 2		+	Rice, KC/d					*	180.2407				*		0.0118	*	0.01165			=		0.0248	
Nondiet 1		+	Vitamin A deficiency in age of <5 years					*	0.2987				*		0.1519	*	0.99036			=		0.0449	
Nondiet 1		+	Child underweight of >2 SD					*	0.3433				*		0.1780	*	0.99036			=		0.0605	
Nondiet 1		+	Ambient pollution, PM_0.25_					*	1.0000				*		0.0752	*	0.99036			=		0.0745	
Nondiet 1		+	Smoking rate (0-1)					*	0.1451				*		0.1866	*	0.99036			=		0.0268	
Nondiet 1		+	Sublingual tobacco rate (0-1)					*	0.1422				*		0.1300	*	0.99036			=		0.0183	
Nondiet 1		+	Blood lead level, mcg/dL					*	1.0000				*		0.0927	*	0.99036			=		0.0918	
Nondiet 1		+	Household air pollution (0-1)					*	0.6660				*		0.1829	*	0.99036			=		0.1207	
Nondiet 1		-	Sociodemographic index (0-1)					*	0.4110				*		0.2680	*	0.99036			=		0.1091	
Sex (m/f)		-	Sex: male, one; female, two					*									0.38437			=		0.3844	
Step 3																							
	Standardized variables						Preliminary risk factor formula							Standardized variables									Final risk factor formula
-	Processed meat, KC/d					*	0.0012				-			Processed meat, KC/d							*		0.05
-	Red meat, KC/d					*	0.0142				-			Red meat, KC/d							*		0.53
-	Fish, KC/d					*	0.0062				-			Fish, KC/d							*		0.23
-	Milk, KC/d					*	0.0209				-			Milk, KC/d							*		0.78
-	Poultry, KC/d					*	0.0527				-			Poultry, KC/d							*		1.98
-	Eggs, KC/d					*	0.0233				-			Eggs, KC/d							*		0.88
-	Added SFA, KC/d					*	0.0597				-			Added SFA, KC/d							*		2.25
-	Added PUFA, KC/d					*	0.0697				-			Added PUFA, KC/d							*		2.62
-	Added TFA, KC/d					*	0.0020				-			Added TFA, KC/d							*		0.07
+	Alcohol, KC/d					*	0.0992				+			Alcohol, KC/d							*		3.73
+	Sugary beverages, KC/d^e^					*	0.0000				+			Sugary beverages, KC/d^e^							*		0.00
-	Potatoes, KC/d					*	0.0070				-			Potatoes, KC/d							*		0.26
-	Sweet potatoes, KC/d^e^					*	0.0041				-			Sweet potatoes, KC/d^e^							*		0.15
-	Corn, KC/d					*	0.0031				-			Corn, KC/d							*		0.12
-	Fruits, KC/d					*	0.0821				-			Fruits, KC/d							*		3.09
-	Vegetables, KC/d					*	0.0135				-			Vegetables, KC/d							*		0.51
-	Nuts and seeds, KC/d					*	0.0011				-			Nuts and seeds, KC/d							*		0.04
-	Whole grains, KC/d					*	0.0050				-			Whole grains, KC/d							*		0.19
-	Legumes, KC/d^e^					*	0.0149				-			Legumes, KC/d^e^							*		0.56
+	Rice, KC/d					*	0.0248				+			Rice, KC/d							*		0.93
+	Vitamin A deficiency in age of <5 years					*	0.0449				+			Vitamin A deficiency in age of <5 years							*		1.69
+	Child underweight of >2 SD					*	0.0605				+			Child underweight of >2 SD							*		2.28
+	Ambient pollution, PM_0.25_					*	0.0745				+			Ambient pollution, PM_0.25_							*		2.80
+	Smoking rate (0-1)					*	0.0268				+			Smoking rate (0-1)							*		1.01
+	Sublingual tobacco rate (0-1)					*	0.0183				+			Sublingual tobacco rate (0-1)							*		0.69
+	Blood lead level, mcg/dL					*	0.0918				+			Blood lead level, mcg/dL							*		3.45
+	Household air pollution (0-1)					*	0.1207				+			Household air pollution (0-1)							*		4.54
-	Sociodemographic index (0-1)					*	0.1091				-			Sociodemographic index (0-1)							*		4.10
-	Sex: male, one; female, two					*	0.3844				-			Sex: male, one; female, two							*		14.46
	Sum						1.4358							Total formula PAR%									54.00
	Sum						1.4358																
	r						0.7349																
	R^2^						0.5400																
	R^2^/sum						0.3761																
	R^2^/sum*100						37.6128																

The derived formula is as follows: NCD risk factor formula from low animal food seven cohorts=-0.05*processed meat-0.53*red meat-0.23*fish-0.78*milk-1.98*poultry-0.88*eggs-2.25*added SFA-2.62*added PUFA-0.07*added TFA+3.73*alcohol+0.00*sugary beverages-0.26*potatoes-0.15*sweet potatoes-0.12*corn-3.09*fruit-0.51*vegetables-0.04*nuts and seeds-0.19*whole grains-0.56*legumes+0.93*rice+1.69*vitamin A deficiency+2.28*severe underweight child+2.80*ambient air pollution+1.01*smoking prevalence+0.69*sublingual tobacco use+3.45*blood lead level+4.54*household air pollution-4.10*socio-demographic index-14.46*sex (male=1; female=2). The total PAR% of all risk factors=54.00%.

Note the similarities and differences of the low animal food seven formulas. All animal food seven and healthy plant seven risk factors in both formulas had negative coefficients. This suggests that they would have had lower risk of early deaths from NCDs with higher consumption of healthy animal and plant foods.

We next modeled an NCD risk factor formula from high animal food seven cohorts including all 500 m/f pairs of cohorts (1000 cohorts) in Table 7 and other cohorts with mean m/f animal food seven of ≥400 KC/d. Table 15 shows the subset basic statistics, and Table 16 gives the two step derivation of the multiple regression risk factor formula.

**Table 15:** High animal food seven subset, n=1722 cohorts and m/f paired NCD, noncommunicable disease; SD, standard deviation; CI, confidence interval; KC/d, kilocalories/day; SFA, saturated fatty acid; PUFA, polyunsaturated fatty acid; TFA, trans fatty acid; METs, metabolic equivalent of tasks; PM_0.25_, particulate matter 0.25; BMI, body mass index; FPG, fasting plasma glucose; LDLc, low-density lipoprotein cholesterol; SBP, systolic blood pressure; SDI, sociodemographic index; m/f, male/female

NCDs versus risk factors, NCDs of <1070.22659 deaths/100000/year or animal food seven of >400 KC/d, foods in KC/d, n=1722 cohorts, and mean m/f	Mean	SD	Minimum	Maximum	r	95% CI low	95% CI high	P
NCD deaths/100000/year	1085	253.94	634.58	2193				
Processed meat, KC/d	16.64	14.71	1.01	55.39	0.093	0.046	0.139	0.0001
Red meat, KC/d	100.38	43.78	14.93	194.65	0.411	0.371	0.450	<0.0001
Fish, KC/d	33.80	72.66	2.89	337.58	-0.434	-0.471	-0.395	<0.0001
Milk, KC/d	63.15	30.82	12.21	135.11	0.243	0.198	0.287	<0.0001
Poultry, KC/d	90.04	43.00	5.75	289.96	0.232	0.186	0.276	<0.0001
Eggs, KC/d	28.41	10.18	5.78	63.43	-0.197	-0.242	-0.151	<0.0001
Added SFA, KC/d	149.58	41.75	70.30	316.45	0.286	0.242	0.329	<0.0001
Added PUFA, KC/d	99.22	46.83	17.95	209.68	0.201	0.155	0.246	<0.0001
Added TFA, KC/d	12.23	9.59	2.20	38.53	0.135	0.088	0.181	<0.0001
Alcohol, KC/d	117.57	68.50	10.28	338.52	-0.007	-0.054	0.040	0.7695
Sugary beverages, KC/d	326.99	222.27	92.46	1392	0.057	0.010	0.104	0.0182
Potatoes, KC/d	88.62	41.77	8.30	287.77	0.348	0.306	0.389	<0.0001
Sweet potatoes, KC/d	4.00	6.82	0.03	98.95	-0.172	-0.218	-0.126	<0.0001
Corn, KC/da	28.47	41.41	0.96	236.88	-0.132	-0.178	-0.085	<0.0001
Fruits, KC/d	64.39	17.32	20.50	150.62	-0.046	-0.093	0.001	0.055
Vegetables, KC/d	102.64	43.45	9.62	287.48	-0.216	-0.260	-0.170	<0.0001
Nuts/seeds, KC/d	15.98	11.63	0.07	98.65	-0.062	-0.109	-0.015	0.0099
Whole grains, KC/d	51.42	27.37	1.64	160.24	-0.098	-0.145	-0.051	<0.0001
Legumes, KC/d	45.26	29.57	3.20	123.96	-0.209	-0.254	-0.163	<0.0001
Rice, KC/d	43.84	46.47	2.93	174.51	-0.382	-0.421	-0.341	<0.0001
Animal food seven, KC/d	482.00	139.91	149.45	737.21	0.109	0.062	0.155	<0.0001
Healthy plant seven, KC/d	382.91	89.23	207.63	710.80	-0.129	-0.175	-0.082	<0.0001
Total available KC/d	3069	357	1948	3898	0.325	0.282	0.367	<0.0001
Vitamin A deficiency in age of <5 years	13006	8455	1475	43817	0.142	0.096	0.188	<0.0001
Sodium, gram/day	3.66	0.94	1.88	6.56	-0.158	-0.204	-0.112	<0.0001
Calcium, gram/day	0.548	0.169	0.198	1.004	0.239	0.194	0.283	<0.0001
Fiber, gram/day	10.56	1.89	5.90	16.70	-0.238	-0.282	-0.193	<0.0001
Physical activity METs	3508	874.56	2015	7496	0.235	0.190	0.279	<0.0001
Child underweight of >2 SD	0.033	0.037	0.006	0.205	-0.294	-0.336	-0.250	<0.0001
Stop breast feeding in <6 months	0.182	0.034	0.072	0.226	0.279	0.235	0.322	<0.0001
Ambient pollution, PM_0.25_	17.28	12.42	4.38	87.22	-0.146	-0.192	-0.099	<0.0001
Smokers (0-1)	0.215	0.066	0.050	0.429	0.234	0.188	0.278	<0.0001
Secondhand smoke (0-1)	0.319	0.056	0.178	0.545	-0.102	-0.149	-0.055	<0.0001
Sublingual tobacco (0-1)	0.013	0.017	0.001	0.064	-0.105	-0.152	-0.058	<0.0001
Blood lead, mcg/dL	4.23	0.90	1.45	6.57	0.067	0.020	0.114	0.0053
Household air pollution (0-1)	0.074	0.139	0.000	0.829	-0.001	-0.048	0.046	0.9714
Kidney disease III (0-1)	0.046	0.019	0.019	0.097	-0.040	-0.087	0.007	0.0966
BMI, kg/m^2^	24.45	1.60	20.20	29.29	0.435	0.396	0.473	<0.0001
FPG, mmol/L	4.57	0.30	3.62	5.35	0.105	0.058	0.151	<0.0001
LDLc, mmol/L	2.79	0.25	1.69	3.24	0.137	0.090	0.183	<0.0001
SBP, mm Hg	133.59	4.39	124.18	145.12	0.009	-0.038	0.056	0.703
SDI (0-1)	0.750	0.132	0.351	0.896	0.105	0.058	0.151	<0.0001

**Table 16:** Multiple regression formula derivation 3: high animal food seven, m/f paired ^d^See Appendices for the methodology of deriving multiple regression risk factor formulas KC/d, kilocalories/day; SFA, saturated fatty acid; PUFA, polyunsaturated fatty acid; TFA, trans fatty acid; SD, standard deviation; PM_0.25_, particulate matter 0.25; PAR%, population-attributable risk percent; m/f, male/female; BMI, body mass index; LDLc, low-density lipoprotein cholesterol; FPG, fasting plasma glucose

Step 1^d^																	
Combination risk factors			Relevant variables standardized				Mean				R^2^		Parameter estimates			Preliminary risk factor formula	
Diet 1		+	Processed meat, KC/d			*	16.6409			*	0.00857	*	0.00672	=		0.00096	
Diet 1		+	Red meat, KC/d			*	100.3818			*	0.16892	*	0.00672	=		0.11395	
Diet 1		-	Fish, KC/d			*	33.7988			*	0.18809	*	0.00672	=		0.04272	
Diet 1		+	Milk, KC/d			*	63.1507			*	0.05889	*	0.00672	=		0.02499	
Diet 1		+	Poultry, KC/d			*	90.0409			*	0.05362	*	0.00672	=		0.03245	
Diet 1		-	Eggs, KC/d			*	28.4076			*	0.03873	*	0.00672	=		0.00739	
Diet 1		+	Added SFA, KC/d			*	149.5187			*	0.08163	*	0.00672	=		0.08202	
Diet 1		+	Added PUFA, KC/d			*	99.3074			*	0.04047	*	0.00672	=		0.02701	
Diet 1		+	Added TFA, KC/d			*	12.1333			*	0.01811	*	0.00672	=		0.00148	
Diet 1		+	Alcohol, KC/d			*	117.5688			*	0.00005	*	0.00672	=		0.00004	
Diet 1		+	Sugary beverages, KC/d			*	326.9846			*	0.00324	*	0.00672	=		0.00711	
Diet 1		+	Potatoes, KC/d			*	88.6168			*	0.12138	*	0.00672	=		0.07228	
Diet 1		-	Sweet potatoes, KC/d			*	4.0046			*	0.02968	*	0.00672	=		0.00080	
Diet 1		-	Corn, KC/d			*	28.4713			*	0.01735	*	0.00672	=		0.00332	
Diet 1		-	Fruits, KC/d			*	64.3867			*	0.00214	*	0.00672	=		0.00093	
Diet 1		-	Vegetables, KC/d			*	102.6415			*	0.04662	*	0.00672	=		0.03215	
Diet 1		-	Nuts/seeds, KC/d			*	15.9814			*	0.00386	*	0.00672	=		0.00041	
Diet 1		-	Whole grains, KC/d			*	51.4225			*	0.00968	*	0.00672	=		0.00335	
Diet 1		-	Legumes, KC/d			*	45.2631			*	0.04362	*	0.00672	=		0.01327	
Diet 1		-	Rice, KC/d			*	43.8431			*	0.14574	*	0.00672	=		0.04294	
Nondiet 1		+	Vitamin A deficiency in age of <5 years			*	0.1301			*	0.02028	*	7.44471	=		0.01964	
Nondiet 1		+	Stop breast feeding in <6 months			*	0.1816			*	0.07780	*	7.44471	=		0.10519	
Nondiet 1		+	Smoking rate (0-1)			*	0.2144			*	0.05455	*	7.44471	=		0.08707	
Nondiet 1		+	Blood lead, mcg/dL			*	1.0000			*	0.00450	*	7.44471	=		0.03350	
Nondiet 1		+	BMI, kg/m^2^			*	1.0000			*	0.18956	*	0.82935	=		0.15722	
Nondiet 1		+	LDLc, mmol/L			*	1.0000				0.01098	*	0.82935	=		0.00911	
Nondiet 1		+	FPG, mmol/L			*	1.0000			*	0.01870		0.82935	=		0.01551	
Step 2																	
	Relevant variables standardized				Preliminary risk factor formula												Final risk factor formula
+	Processed meat, KC/d			*	0.00096			+	Processed meat, KC/d						*		0.03
+	Red meat, KC/d			*	0.11395			+	Red meat, KC/d						*		3.51
-	Fish, KC/d			*	0.04272			-	Fish, KC/d						*		1.31
+	Milk, KC/d			*	0.02499			+	Milk, KC/d						*		0.77
+	Poultry, KC/d			*	0.03245			+	Poultry, KC/d						*		1.00
-	Eggs, KC/d			*	0.00739			-	Eggs, KC/d						*		0.23
+	Added SFA, KC/d			*	0.08202			+	Added SFA, KC/d						*		2.52
+	Added PUFA, KC/d			*	0.02701			+	Added PUFA, KC/d						*		0.83
+	Added TFA, KC/d			*	0.00148			+	Added TFA, KC/d						*		0.05
+	Alcohol, KC/d			*	0.00004			+	Alcohol, KC/d						*		0.00
+	Sugary beverages, KC/d			*	0.00711			+	Sugary beverages, KC/d						*		0.22
+	Potatoes, KC/d			*	0.07228			+	Potatoes, KC/d						*		2.22
-	Sweet potatoes, KC/d			*	0.00080			-	Sweet potatoes, KC/d						*		0.02
-	Corn, KC/d			*	0.00332			-	Corn, KC/d						*		0.10
-	Fruits, KC/d			*	0.00093			-	Fruits, KC/d						*		0.03
-	Vegetables, KC/d			*	0.03215			-	Vegetables, KC/d						*		0.99
-	Nuts/seeds, KC/d			*	0.00041			-	Nuts/seeds, KC/d						*		0.01
-	Whole grains, KC/d			*	0.00335			-	Whole grains, KC/d						*		0.10
-	Legumes, KC/d			*	0.01327			-	Legumes, KC/d						*		0.41
-	Rice, KC/d			*	0.04294			-	Rice, KC/d						*		1.32
+	Vitamin A deficiency in age of <5 years			*	0.01964			+	Vitamin A deficiency in age of <5 years						*		0.60
+	Stop breast feeding in <6 months			*	0.10519			+	Stop breast feeding in <6 months						*		3.24
+	Smoking rate (0-1)			*	0.08707			+	Smoking rate (0-1)						*		2.68
+	Blood lead, mcg/dL			*	0.03350			+	Blood lead, mcg/dL						*		1.03
+	BMI, kg/m^2^			*	0.15722			+	BMI, kg/m^2^						*		4.84
+	LDLc, mmol/L			*	0.00911			+	LDLc, mmol/L						*		0.28
+	FPG, mmol/L			*	0.01551			+	FPG, mmol/L						*		0.48
	Sum				0.93679				Total formula PAR%								28.83
	Sum				0.93679												
	r				0.53692												
	R^2^				0.28828												
	R^2^/sum				0.30774												
	Final formula=preliminary formula*R^2^/sum*100				30.77350												

To compare with the low animal food formulas, the high animal food formula with paired male and female cohorts is as follows: NCD risk factor formula for high animal food seven cohorts=0.03*processed meat+3.51*red meat-1.31*fish+0.77*milk+1.00*poultry-0.23*eggs+2.52*added SFA+0.83*added PUFA+0.05*added TFA+0.00*alcohol+0.22*sugary beverages+2.22*potatoes-0.02*sweet potatoes-0.10*corn-0.03*fruit-0.99*vegetables-0.01*nuts and seeds-0.10*whole grains-0.41*legumes-1.32*rice+0.60*vitamin A deficiency in children+3.24*stopping breast feeding before six months+2.68*smoking prevalence+1.03*lead+4.84*body mass index+0.28*low-density lipoprotein cholesterol+0.48*fasting plasma glucose. The total PAR% of all risk factors=28.83%.

In contrast to Table 15 with mean values of male/female cohorts, Table 17 shows the high animal food seven subset with individual male and female cohorts.

**Table 17:** High animal food seven subset: n=1722 cohorts, m/f not paired NCD, noncommunicable disease; SD, standard deviation; CI, confidence interval; KC/d, kilocalories/day; SFA, saturated fatty acid; PUFA, polyunsaturated fatty acid; TFA, trans fatty acid; METs, metabolic equivalent of tasks; PM_0.25_, particulate matter 0.25; BMI, body mass index; LDLc, low-density lipoprotein cholesterol; SBP, systolic blood pressure; FPG, fasting plasma glucose

NCD deaths/100000/year versus risk factors, NCDs of <1070.22659 or animal food seven of >400 KC/d, and n=1722 cohorts	Mean	SD	Minimum	Maximum	r	95% CI low	95% CI high	P
NCD deaths/100000/year	1085.00	397.73	423.80	3021.00				
Processed meat, KC/d	16.64	15.49	0.87	68.77	0.192	0.146	0.237	<0.0001
Red meat, KC/d	100.38	49.34	12.03	235.95	0.562	0.529	0.593	<0.0001
Fish, KC/d	33.80	73.02	2.66	370.36	-0.249	-0.293	-0.204	<0.0001
Milk, KC/d	63.15	30.86	11.93	146.82	0.180	0.134	0.225	<0.0001
Poultry, KC/d	90.04	43.00	5.75	289.96	0.148	0.101	0.194	<0.0001
Eggs, KC/d	28.41	10.18	5.78	63.43	-0.126	-0.172	-0.079	<0.0001
Added SFA, KC/d	149.52	43.46	64.46	342.63	0.370	0.328	0.410	<0.0001
Added PUFA, KC/d	99.31	48.01	16.25	229.82	0.262	0.218	0.306	<0.0001
Added TFA, KC/d	12.13	9.51	2.15	38.98	0.076	0.028	0.122	0.0017
Alcohol, KC/d	117.57	74.26	4.25	429.81	0.242	0.197	0.286	<0.0001
Sugary beverages, KC/d	326.98	231.44	72.91	1472.00	0.185	0.139	0.230	<0.0001
Potatoes, KC/d	88.62	41.77	8.30	287.77	0.222	0.177	0.267	<0.0001
Sweet potatoes, KC/d	4.00	6.82	0.03	98.95	-0.110	-0.156	-0.063	<0.0001
Corn, KC/d	28.47	41.41	0.96	236.88	-0.084	-0.131	-0.037	0.0005
Fruits, KC/d	64.39	17.89	19.20	161.39	-0.186	-0.232	-0.140	<0.0001
Vegetables, KC/d	102.64	43.84	9.48	304.17	-0.083	-0.130	-0.036	0.0005
Nuts and seeds, KC/d	15.98	11.66	0.07	102.99	-0.010	-0.057	0.037	0.6773
Whole grains, KC/d	51.42	27.52	1.61	166.79	-0.023	-0.071	0.024	0.3307
Legumes, KC/d	45.26	29.89	2.95	134.74	-0.061	-0.108	-0.014	0.0113
Rice, KC/d	43.84	46.47	2.93	174.51	-0.244	-0.288	-0.199	<0.0001
Animal food seven, KC/d	481.94	145.97	135.90	794.80	0.268	0.224	0.312	<0.0001
Healthy plant seven, KC/d	383.01	90.80	203.52	748.17	0.025	-0.022	0.072	0.3024
Total KC/d	3069	357.38	1948	3898	0.208	0.162	0.252	<0.0001
Vitamin A deficiency in age of <5 years	13006	8487	1368	44100	0.072	0.025	0.119	0.0027
Sodium, gram/day	3.66	0.96	1.33	6.70	0.010	-0.038	0.057	0.6899
Calcium, gram/day	0.55	0.17	0.19	1.04	0.273	0.229	0.317	<0.0001
Dietary fiber, gram/day	10.56	2.06	5.41	18.15	0.106	0.059	0.152	<0.0001
Physical activity METs	3508	1024	1609	7607	0.473	0.435	0.509	<0.0001
Child underweight of >2 SD	0.03	0.04	0.00	0.24	-0.167	-0.213	-0.121	<0.0001
Stop breast feeding in <6 months	0.18	0.03	0.07	0.23	0.178	0.132	0.224	<0.0001
Ambient pollution, PM_0.25_	17.28	12.42	4.38	87.22	-0.093	-0.140	-0.046	0.0001
Smoking rate (0-1)	0.21	0.10	0.01	0.48	0.459	0.421	0.496	<0.0001
Secondhand smoking (0-1)	0.32	0.08	0.16	0.65	-0.234	-0.278	-0.189	<0.0001
Sublingual tobacco (0-1)	0.01	0.02	0.00	0.12	0.161	0.115	0.207	<0.0001
Blood lead, mcg/dL	4.23	0.96	1.22	7.20	0.215	0.170	0.260	<0.0001
Household air pollution (0-1)	0.07	0.14	0.00	0.84	-0.001	-0.048	0.046	0.9674
Kidney disease stage III (0-1)	0.05	0.02	0.02	0.12	-0.242	-0.285	-0.197	<0.0001
BMI, kg/m^2^	24.45	1.65	19.61	29.39	0.283	0.239	0.326	<0.0001
LDLc, mmol/L	2.79	0.26	1.60	3.25	0.027	-0.021	0.074	0.2702
FPG, mmol/L	4.57	0.31	3.54	5.58	0.029	-0.019	0.076	0.2337
SBP, mm Hg	133.59	4.58	123.41	146.00	0.130	0.083	0.176	<0.0001
Sociodemographic index (0-1)	0.75	0.13	0.35	0.90	0.067	0.020	0.114	0.0055
Sex: male, one; female, two	1.50	0.50	1.00	2.00	-0.688	-0.712	-0.662	<0.0001

From the above Table 17 subset, Table 18 shows the two-step derivation of the high animal food seven multiple regression risk factor formula.

**Table 18:** Multiple regression formula two-step derivation 4: low animal food seven, m/f unpaired ^d^See Appendices for the methodology of deriving multiple regression risk factor formulas KC/d, kilocalories/day; SFA, saturated fatty acid; PUFA, polyunsaturated fatty acid; TFA, trans fatty acid; SD, standard deviation; PAR%, population-attributable risk percent; m/f, male/female; BMI, body mass index; FPG, fasting plasma glucose; SBP, systolic blood pressure; m/f, male/female

Step 1^d^																			
Combination risk factors				Standardized variables				Mean			R^2^				Parameter estimates		Preliminary risk factor formula		
Diet 1		+		Processed meat, KC/d			*	16.6409		*	0.0368			*	0.00263	=	0.00161		
Diet 1		+		Red meat, KC/d			*	100.3818		*	0.3158			*	0.00263	=	0.08337		
Diet 1		-		Fish, KC/d			*	33.7988		*	0.0621			*	0.00263	=	0.00552		
Diet 1		+		Milk, KC/d			*	63.1507		*	0.0323			*	0.00263	=	0.00537		
Diet 1		+		Poultry, KC/d			*	90.0409		*	0.0219			*	0.00263	=	0.00518		
Diet 1		-		Eggs, KC/d			*	28.4076		*	0.0158			*	0.00263	=	0.00118		
Diet 1		+		Added SFA, KC/d			*	149.5187		*	0.1368			*	0.00263	=	0.05378		
Diet 1		+		Added PUFA, KC/d			*	99.3074		*	0.0688			*	0.00263	=	0.01796		
Diet 1		+		Added TFA, KC/d			*	12.1333		*	0.0057			*	0.00263	=	0.00018		
Diet 1		+		Alcohol, KC/d			*	117.5688		*	0.0585			*	0.00263	=	0.01808		
Diet 1		+		Sugary beverages, KC/d			*	326.9846		*	0.0343			*	0.00263	=	0.02952		
Diet 1		+		Potatoes, KC/d			*	88.6168		*	0.0495			*	0.00263	=	0.01154		
Diet 1		-		Sweet potatoes, KC/d			*	4.0046		*	0.0121			*	0.00263	=	0.00013		
Diet 1		-		Corn, KC/d			*	28.4713		*	0.0071			*	0.00263	=	0.00053		
Diet 1		-		Fruits, KC/d			*	64.3867		*	0.0348			*	0.00263	=	0.00589		
Diet 1		-		Vegetables, KC/d			*	102.6415		*	0.0070			*	0.00263	=	0.00188		
Diet 1		-		Nuts and seeds, KC/d			*	15.9814		*	0.0001			*	0.00263	=	0.00000		
Diet 1		-		Whole grains, KC/d			*	51.4225		*	0.0006			*	0.00263	=	0.00007		
Diet 1		-		Legumes, KC/d			*	45.2631		*	0.0037			*	0.00263	=	0.00044		
Diet 1		-		Rice, KC/d			*	43.8431		*	0.0594			*	0.00263	=	0.00685		
Nondiet 1		+		Vitamin A deficiency in age of <5 years			*	0.1301		*	0.0052			*	1.28741	=	0.00087		
Nondiet 1		+		Stop breast feeding in <6 months			*	0.1816		*	0.0318			*	1.28741	=	0.00743		
Nondiet 1		+		Smoking rate (0-1)			*	0.2144		*	0.2111			*	1.28741	=	0.05827		
Nondiet 1		+		Sublingual tobacco (0-1)			*	0.0130		*	0.0261			*	1.28741	=	0.00044		
Nondiet 1		+		Sociodemographic index (0-1)			*	0.7503		*	0.0045			*	1.28741	=	0.00432		
Nondiet 1		+		Blood lead, mcg/dL			*	1.0000		*	0.0464			*	1.28741	=	0.05975		
Nondiet 2		+		BMI, kg/m^2^			*	1.0000		*	0.0799			*	0.10480	=	0.00837		
Nondiet 2		+		SBP, mm Hg			*	1.0000		*	0.0168			*	0.10480	=	0.00176		
Sex		-		Sex: male, one; female, two			*							*	0.47310	=	0.47310		
Step 2																			
Combination risk factors			Standardized variables			Preliminary risk factor formula							Standardized variables						Final risk factor formula
Diet 1	+		Processed meat, KC/d		*	0.00161			Diet 1			+	Processed meat, KC/d					*	0.10
Diet 1	+		Red meat, KC/d		*	0.08337			Diet 1			+	Red meat, KC/d					*	5.38
Diet 1	-		Fish, KC/d		*	0.00552			Diet 1			-	Fish, KC/d					*	0.36
Diet 1	+		Milk, KC/d		*	0.00537			Diet 1			+	Milk, KC/d					*	0.35
Diet 1	+		Poultry, KC/d		*	0.00518			Diet 1			+	Poultry, KC/d					*	0.33
Diet 1	-		Eggs, KC/d		*	0.00118			Diet 1			-	Eggs, KC/d					*	0.08
Diet 1	+		Added SFA, KC/d		*	0.05378			Diet 1			+	Added SFA, KC/d					*	3.47
Diet 1	+		Added PUFA, KC/d		*	0.01796			Diet 1			+	Added PUFA, KC/d					*	1.16
Diet 1	+		Added TFA, KC/d		*	0.00018			Diet 1			+	Added TFA, KC/d					*	0.01
Diet 1	+		Alcohol, KC/d		*	0.01808			Diet 1			+	Alcohol, KC/d					*	1.17
Diet 1	+		Sugary beverages, KC/d		*	0.02952			Diet 1			+	Sugary beverages, KC/d					*	1.91
Diet 1	+		Potatoes, KC/d		*	0.01154			Diet 1			+	Potatoes, KC/d					*	0.75
Diet 1	-		Sweet potatoes, KC/d		*	0.00013			Diet 1			-	Sweet potatoes, KC/d					*	0.01
Diet 1	-		Corn, KC/d		*	0.00053			Diet 1			-	Corn, KC/d					*	0.03
Diet 1	-		Fruits, KC/d		*	0.00589			Diet 1			-	Fruits, KC/d					*	0.38
Diet 1	-		Vegetables, KC/d		*	0.00188			Diet 1			-	Vegetables, KC/d					*	0.12
Diet 1	-		Nuts and seeds, KC/d		*	0.00000			Diet 1			-	Nuts and seeds, KC/d					*	0.00
Diet 1	-		Whole grains, KC/d		*	0.00007			Diet 1			-	Whole grains, KC/d					*	0.00
Diet 1	-		Legumes, KC/d		*	0.00044			Diet 1			-	Legumes, KC/d					*	0.03
Diet 1	-		Rice, KC/d		*	0.00685			Diet 1			-	Rice, KC/d					*	0.44
Nondiet 1	+		Vitamin A deficiency in age of <5 years		*	0.00087			Nondiet 1			+	Vitamin A deficiency in age of <5 years					*	0.06
Nondiet 1	+		Stop breast feeding in <6 months		*	0.00743			Nondiet 1			+	Stop breast feeding in <6 months					*	0.48
Nondiet 1	+		Smoking rate (0-1)		*	0.05827			Nondiet 1			+	Smoking rate (0-1)					*	3.76
Nondiet 1	+		Sublingual tobacco (0-1)		*	0.00044			Nondiet 1			+	Sublingual tobacco (0-1)					*	0.03
Nondiet 1	+		Sociodemographic index (0-1)		*	0.00432			Nondiet 1			+	Sociodemographic index (0-1)					*	0.28
Nondiet 1	+		Blood lead, mcg/dL		*	0.05975			Nondiet 1			+	Blood lead, mcg/dL					*	3.86
Nondiet 2	+		BMI, kg/m^2^		*	0.00837			Nondiet 2			+	BMI, kg/m^2^					*	0.54
Nondiet 2	+		SBP, mm Hg		*	0.00176			Nondiet 2			+	SBP, mm Hg					*	0.11
Sex (m/f)	-		Sex: male, one; female, two		*	0.47310			Sex (m/f)			-	Sex: male, one; female, two					*	30.55
			Sum			0.86341							Total formula PAR%						55.76
			Sum			0.86341													
			r			0.74672													
			R^2^			0.55759													
			R^2^/sum			0.64580													
			Final formula=R^2^/sum*100*preliminary formula			64.5800													

The resulting high animal food seven multiple regression formula is as follows: NCD risk factor formula for individual male and female high animal food seven cohorts=0.10*processed meat+5.38*red meat-0.36*fish+0.35*milk+0.33*poultry-0.08*eggs+3.47*added SFA+1.16*added PUFA+0.01*added TFA+1.17*alcohol+1.91*sugary beverages+0.75*potatoes-0.01*sweet potatoes-0.03*corn-0.38*Fruit-0.12*vegetables-0.00*nuts and seeds-0.00*whole grains-0.03*legumes-0.44*rice+0.06*vitamin A deficiency in children+0.48*stopping breast feeding before six months+3.76*smoking prevalence+0.03*sublingual tobacco use+3.86*lead+0.28*sociodemographic index+0.54*body mass index+0.11*systolic blood pressure-30.55*sex (male=1; female=2). The total PAR% of risk factors=55.76%.

Deriving multiple regression risk factor formulas with mean risk factor values from male and female cohorts eliminated the dominant role of sex in some of the PAR% values. The difference between the total formula PAR% with m/f mean values and unpaired risk factors was accounted for by sex differences. Illustrating this factor, males worldwide had much higher NCDs and were exposed to more meat than females (e.g., mean red meat: males=60.7 KC/d; females=39.9 KC/d), alcohol (means: males=105.8 KC/d; females=56.3 KC/d), smoking tobacco (means: males=34.0%; females=7.0%), and lead (means: males=5.35 mcg/dL; females=4.67 mcg/dL). Conversely, males consumed less fruit (fruit means: males=37.6 KC/d, and females=42.8 KC/d). Major differences between exposures of males and females affected the multiple regression formula when males and females were unpaired but not when combined as mean values.

Comparing Planetary Health Diet (PHD) recommendations with GBD data

In what is analogous to the 22 dietary risk factors with GBD data, EAT [1] published the PHD animal- and plant-based foods (KC/d) recommended ranges. To compare GBD data with the PHD, we began with the mean m/f values from Table 11 for the lower ranges’ boundaries (NCDs of <1070.2 deaths/100000/year and animal food seven of <400 KC/d {n=416 cohorts} or mean m/f animal food seven of <149 KC/d {animal food seven of Kenya}, n=2724 cohorts). For the upper ranges’ boundaries, we used the high animal food seven subset (Table 13: NCDs of <1070.2 deaths/100000/year {n=1000 cohorts in Table 7} or animal food seven of ≥400 KC/d {n=722 additional cohorts}, total n=1722 cohorts). From the methodology detailed in Appendices, Table 19 shows the three-step derivation of estimates of the optimal ranges for 22 dietary risk factors, as well as the PHD KC/d suggested dietary ranges of 14 dietary risk factors.

**Table 19:** GBD versus PHD estimates for the optimal risk factor ranges (KC/d and m/f means) ^f^See Appendices for the methodology of deriving optimal food ranges to minimize NCDs NCD, noncommunicable disease; KC/d, kilocalories/day; SFA, saturated fatty acid; PUFA, polyunsaturated fatty acid; TFA, trans fatty acid; NA, not available; m/f, male/female

Step 1^f^												
Table 11: NCDs of <1070.22659 deaths/100000/year or animal food seven of <149 KC/d, n=2724 cohorts, and mean m/f		Mean		r			R^2^		Multiplier=1±R^2^ depending on the sign of the r: + if r is - and - if r is +		Multiplier*diet variable means=preliminary lower limits of optimal ranges	
Processed meat, KC/d		1.46		-0.399			0.159		1.159		1.69	
Red meat, KC/d		17.01		-0.445			0.198		1.198		20.37	
Fish, KC/d		2.52		-0.610			0.372		1.372		3.45	
Milk, KC/d		15.98		-0.427			0.183		1.183		18.89	
Poultry, KC/d		17.53		-0.614			0.377		1.377		24.13	
Eggs, KC/d		7.07		-0.643			0.413		1.413		9.99	
Added SFA, KC/d		85.54		-0.465			0.216		1.216		104.04	
Animal food seven, KC/d		147.09		-0.667			0.445		1.445		212.49	
Added PUFA, KC/d		27.62		-0.627			0.394		1.394		38.49	
Added TFA, KC/d		9.15		-0.150			0.023		1.023		9.36	
Alcohol, KC/d		77.05		0.132			0.017		0.983		75.70	
Sugary beverages, KC/d		315.61		0.431			0.186		0.814		256.99	
Potatoes, KC/d		83.22		-0.103			0.011		1.011		84.09	
Sweet potatoes, KC/d		14.33		-0.052			0.003		1.003		14.37	
Corn, KC/d		45.13		-0.092			0.008		1.008		45.52	
Fruits, KC/d		32.97		-0.514			0.264		1.264		41.67	
Vegetables, KC/d		64.20		-0.199			0.040		1.040		66.75	
Nuts/seeds, KC/d		5.77		-0.176			0.031		1.031		5.95	
Whole grains, KC/d		59.54		-0.148			0.022		1.022		60.84	
Legumes, KC/d		74.47		-0.025			0.001		1.001		74.52	
Rice, KC/d		180.24		0.131			0.017		0.983		179.26	
Healthy plant seven, KC/d		278.90		-0.474			0.225		1.225		341.52	
Step 2												
Table 15: NCDs of <1070.22659 deaths/100000/year or animal food seven of >400 KC/d, n=1722 cohorts, and mean m/f		Mean		r			R^2^		Multiplier=1±R^2^ depending on the sign of the r: + if r is - and - if r is +		Multiplier*diet variable means=preliminary upper limits of optimal ranges	
Processed meat, KC/d		16.64		0.192			0.037		0.963		16.03	
Red meat, KC/d		100.38		0.562			0.316		0.684		68.68	
Fish, KC/d		33.80		-0.249			0.062		1.062		35.90	
Milk, KC/d		63.15		0.180			0.032		0.968		61.11	
Poultry, KC/d		90.04		0.148			0.022		0.978		88.07	
Eggs, KC/d		28.41		-0.126			0.016		1.016		28.86	
Added SFA, KC/d		149.52		0.370			0.137		0.863		129.07	
Animal food seven, KC/d		481.94		0.269			0.072		0.928		447.19	
Added PUFA, KC/d		99.31		0.262			0.069		0.931		92.48	
Added TFA, KC/d		12.13		0.076			0.006		0.994		12.06	
Alcohol, KC/d		117.57		0.242			0.058		0.942		110.69	
Sugary beverages, KC/d		326.98		0.185			0.034		0.966		315.76	
Potatoes, KC/d		88.62		0.223			0.050		0.950		84.23	
Sweet potatoes, KC/d		4.00		-0.110			0.012		1.012		4.05	
Corn, KC/d		28.47		-0.084			0.007		1.007		28.67	
Fruits, KC/d		64.39		-0.186			0.035		1.035		66.62	
Vegetables, KC/d		102.64		-0.084			0.007		1.007		103.36	
Nuts/seeds, KC/d		15.98		-0.010			0.000		1.000		15.98	
Whole grains, KC/d		51.42		-0.023			0.001		1.001		51.45	
Legumes, KC/d		45.26		-0.061			0.004		1.004		45.43	
Rice, KC/d		43.84		-0.244			0.059		1.059		46.45	
Healthy plant seven, KC/d		383.01		0.025			0.001		0.999		382.77	
Step 3												
Global Burden of Disease (GBD) food risk factors’ optimal range estimates, KC/d	Lower boundaries for optimal range for food risk factors, KC/d		Means of lower and upper risk factor boundary values		Upper boundaries for optimal range for food risk factors, KC/d	Planetary Health Diet (PHD) risk factors’ KC/d optimal range estimates		Lower boundaries for optimal range for food risk factors, KC/d		Means of lower and upper risk factor boundary values		Upper boundaries for optimal range for food risk factors, KC/d
Processed meat, KC/d	1.69		8.86		16.03	NA						
Red meat, KC/d	20.37		44.52		68.68	Beef, lamb, and pork, KC/d		0		30		60
Fish, KC/d	3.45		19.68		35.90	Fish, KC/d		0		40		143
Milk, KC/d	18.89		40.00		61.11	Dairy, KC/d		0		153		306
Poultry, KC/d	24.13		56.10		88.07	Poultry, KC/d		0		62		124
Eggs, KC/d	9.99		19.42		28.86	Eggs, KC/d		0		19		37
Added SFA, KC/d	104.04		116.55		129.07	Saturated oils, KC/d		0		96		96
Animal food seven, KC/d	212.49		329.84		447.19	Animal food seven, KC/d		0		400		400
Added PUFA, KC/d	38.49		65.49		92.48	Unsaturated oils, KC/d		177		354		708
Added TFA, KC/d	9.36		10.71		12.06	NA						
Alcohol, KC/d	75.70		93.20		110.69	NA						
Sugary beverages, KC/d	256.99		286.37		315.76	All added sugars, KC/d		0		120		120
Potatoes, KC/d	75.75		84.16		92.58	Tubers or starchy vegetables, KC/d		0		39		78
Sweet potatoes, KC/d	4.05		9.21		14.37	NA						
Corn, KC/d	28.67		37.09		45.52	NA						
Fruits, KC/d	41.67		54.15		66.62	Fruits, KC/d		63		126		189
Vegetables, KC/d	66.75		85.05		103.36	Vegetables, KC/d		52		78		156
Nuts/seeds, KC/d	5.95		10.97		15.98	Nuts, KC/d		0		291		437
Whole grains, KC/d	50.53		56.14		61.76	Whole grains, KC/d		811		811		811
Legumes, KC/d	45.43		59.93		74.43	Legumes, KC/d		0		284		426
Rice, KC/d	46.45		112.86		177.13	NA						
Healthy plant seven, KC/d	341.52		362.14		382.77	NA						

In Table 19, the optimal range of animal food seven was 212.5-447.2 KC/d. Cohorts below 212.5 KC/d had very high NCDs, and animal food seven (KC/d) strongly negatively correlated with NCDs (NCDs mean=1554 deaths/100000/year; for NCDs correlated with animal foods seven, r=-0.387, 95% CI=-0.413 to -0.360, p=<0.0001, and n=3944 cohorts). This suggested that more animal food consumption could reduce NCD risk. Cohorts above 447.2 KC/d had relatively low NCDs; however, animal food seven (KC/d and mean m/f) positively correlated with NCDs (NCD mean=1063 deaths/100000/year, and NCDs positively correlated with animal foods 7: r=0.115, 95% CI=0.055-0.174, p<0.0002, and n=1050 cohorts). This suggested that less animal food consumption, other factors being equal, would reduce NCD risk. Less animal food seven would also decrease the risks of common cancers (Table 8 and Table 9).

## Discussion

The EAT-Lancet Commissioners [2] designated red meat as a detrimental food item for which worldwide consumption should be reduced by more than 50%. Table 19 indicated more nuanced health effects of red meat and processed meat than simply being detrimental at any consumption level. These GBD data suggested that minimizing NCDs in developing countries with low animal food seven requires dramatically increasing meat production and consumption. Conversely, reducing meat consumption in high-meat-eating, wealthy countries such as the United States (mean m/f red meat=138.72 KC/d, and mean m/f processed meat=39.90 KC/d, Table 9) would associate with lower NCDs.

Since the PHD lower boundaries for all animal foods were zero, GBD data did not support the PHD recommendation that humans can thrive on a lifelong vegan diet. There has never been a documented case of a human living into old age without ever eating animal-sourced foods. Evolutionary biologist Katharine Milton [13] persuasively maintained that humans could not have satisfied the high nutritional and metabolic demands required to develop a highly evolved, large brain without meat. This is not to say that adopting a vegan diet to counteract overweight or obesity with the associated metabolic and other complications would be inappropriate.

Table 19 shows that the GBD fish consumption optimum range mean (19.68 KC/d) almost doubled the mean consumption of fish worldwide (9.99 KC/d, Table 5). The upper boundary of the fish optimal ranges with GBD data and the PHD recommendation are similar (GBD: 35.90 KC/d versus PHD: 40 KC/d, Table 19). In any case, it will be challenging to even double worldwide fish consumption. About 60% of world fish stocks are fully fished, more than 30% are overfished, and catch by global marine fisheries has been declining since 1996. In addition, a rapidly expanding aquaculture sector can negatively affect coastal habitats and freshwater and terrestrial systems (related to the area directly used for aquaculture and feed production) [14]. To improve human health and reduce NCDs, environmentally regenerating methods of fish farming and aquaculture should be sought.

It would take increasing the consumption of milk-derived products by over sixfold worldwide to achieve the PHD 2050 mean milk recommendation of 153 KC/d (Table 19). The 1990-2017 worldwide mean per capita milk consumption is 25.04 KC/d (Table 5). While increasing global milk output sixfold, it would be practically impossible to halve global processed and red meat consumption (recommended in the PHD). However, with the GBD optimum range mean m/f milk consumption being 40.00 KC/d (Table 19), GBD data suggest that significantly increasing worldwide dairy cow milk production with additional cows going predominantly to developing countries may reduce global NCDs. Countries with mean m/f milk production and consumption greater than the upper boundary of the GBD optimal range (mean m/f milk of >61.11 KC/d, Table 19) might want to reduce milk production, which has been proposed in some European countries based on greenhouse gas emissions [15].

Except for dairy food consumption, the comparisons of GBD with PHD optimal dietary ranges of animal food seven in Table 19 show a significant degree of concordance in orders of magnitude of the mean and upper boundary values for (1) processed meat+red meat/beef, lamb, and pork; (2) fish; (3) poultry; (4) eggs; (5) added SFA; and (6) animal food seven.

With the GBD animal food seven optimal range of 212.49-447.19 KC/d (Table 19) and 20 low-NCD countries with <400 KC/d animal food seven consumption (Table 8), GBD data support the EAT-Lancet Commission’s contention that >400 KC/d of animal food seven is not required for optimal human health. Indeed, early deaths from common cancers were much lower in cohorts with <400 KC/d than cohorts with animal foods of ≥400 KC/d (Table 8 and Table 9).

The amounts of sugary beverages in the GBD optimal range (256.99-315.76 KC/d) was clearly not optimal. The PHD recommendation of 0-120 KC/d for all added sugar would be better for global health but probably not practical. The low global price of sugar ($0.214/pound in February 2023; one pound of sugar contains 1864 KC [16]) suggests that sugar is replacing healthy foods especially in poor countries. Compared with the rest of the world (mean m/f sugary beverages=298.36 KC/d, Table 5), the United States had relatively low sugary beverage intake from 1990 to 2017 (mean sugary beverages=157.20 KC/d). Fruit juices were excluded from fruits by definition (Table 1 [6]), so fruit juices count as sugary beverages.

The PHD-recommended optimal dietary range for tubers/starchy vegetables (0-78 KC/d, mean of 39 KC/d [1]) was low compared with GBD potatoes alone (range: 75.75-92.58 KC/d; mean: 84.16 KC/d, Table 19). The large reduction in starchy vegetable intake recommended in the PHD appeared to be based on prospective observational studies by the Harvard Department of Nutrition [17,18] that showed potatoes are associated with an increased risk of type 2 diabetes and hypertension among US health professionals. However, half or more of the potatoes consumed worldwide were in the form of ultra-processed food products [11]. Data from 79 high- and middle-income countries showed that ultra-processed products dominate the food supplies of high-income countries and that their consumption is now rapidly increasing in middle-income countries [19]. Indeed, recent large prospective observational studies have found higher consumption of ultra-processed foods including potatoes associated with an increased risk of cardiovascular disease incidence and mortality [20].

Maillot and associates [21] found in an econometric evaluation of food groups that “Starches and grains were unique because they were low in disqualifying nutrients yet provided low-cost dietary energy.” Headey and Alderman [22] found that “In lower-income countries, healthy foods were generally expensive, especially most animal-sourced foods.” Given the low cost of starchy vegetables relative to animal foods, fruits, vegetables, and nuts and seeds, there would seem to be no reason to severely reduce starchy vegetable consumption (including minimally processed potatoes) worldwide.

In low sociodemographic index countries (mean SDI=0.410, Table 11), potatoes correlated negatively with NCDs (mean potatoes=83.22 KC/d {r, -0.103; 95% CI, -0.140 to -0.065; p<0.0001}). The Table 12-derived low animal food multiple regression risk factor formula showed that potatoes accounted for PAR% of -0.45% of the NCDs or prevented about seven early deaths/100000/year (mean NCDs=1545; 1545*-0.0045=-6.95 early deaths/100000/year).

However, Table 15 shows that in high sociodemographic index countries (mean SDI=0.750) with similar mean consumption of potatoes, potatoes correlated moderately strongly positively with NCDs (mean potatoes=88.62 KC/d {r: 0.348, 95% CI: 0.306-0.389, and p<0.0001}). The Table 16 multiple regression risk factor formula showed that potatoes accounted for PAR% of 2.22% of the NCDs or about 24 early deaths/100000/year (mean NCDs=1085; 1085*0.0222=24.08 early deaths/100000/year). This supports that the ultra-processing of potatoes in developed countries has a substantial effect in increasing early deaths from NCDs [11].

The PHD proposed (Table 19) radically increasing the production and consumption of the following: (1) nuts, GBD mean m/f nuts and seeds of 10.97 KC/d (range: 5.95-15.98 KC/d, Table 19) versus the PHD mean nut recommendation of 291 KC/d (range: 0-437 KC/d by 2050 [1]) (Table 19); (2) whole grains, GBD global mean m/f whole grains of 56.14 KC/d (range: 50.53-61.76 KC/d, Table 19) versus the PHD recommendation of 811 KC/d by 2050 [1] (Table 19); and (3) legumes, GBD mean m/f legumes of 59.93 KC/d (range: 45.43-74.43 KC/d, Table 19) versus the PHD recommendation of 284 KC/d by 2050 [1] (Table 19).

This analysis shows that these crop increases would not be practical from a worldwide farming perspective and would not be necessary to minimize NCDs. If the global population moved animal food seven consumption into the GBD optimal range to minimize NCDs (212.49-447.19 KC/d), the global animal food consumption would probably not increase above the worldwide mean animal food seven consumption from 1990 to 2017 (worldwide mean animal food seven=254.66 KC/d, Table 5).

The PHD recommended doubling of fruit consumption’s KC/d (mean fruits=126 KC/d {range: 63-189 KC/d}, Table 19) by 2050 [1]. This was much higher than GBD estimates (optimal mean fruit=54.15 KC/d; range: 41.67-66.62 KC/d, Table 19). However, this GBD mean fruit value exceeded the GBD’s 1990-2017 mean (40.21 KC/d) by about 35% (54.15 KC/d/40.21 KC/d=1.35). With a major shift from conventional monocrop farming to regenerative/organic farming scattered widely and close to communities, perhaps fruit production could be tripled by 2050 as recommended by the PHD.

The PHD-recommended mean vegetable consumption by 2050 of 78 KC/d (range: 52-156 KC/d, Table 19) was similar to the GBD optimal range for vegetables (GBD mean vegetables: 85.05 KC/d; range: 66.75-103.36 KC/d). This would not have been a doubling of worldwide vegetables as suggested by the EAT-Lancet Commissioners [1], since the GBD global average of vegetable consumption from 1990 to 2017 was 79.76 KC/d (Table 5).

The global average rice intake was 152.00 KC/d (Table 5). In Table 11, with mean animal food seven of 147.09 KC/d and healthy plant seven of 278.90 KC/d, rice (mean rice=180.24 KC/d) correlated positively with NCDs (r, 0.131; 95% CI, 0.094-0.168; p<0.0001). This might be explained by relatively inexpensive rice substituting for more expensive healthy animal and plant foods in poor countries. It might also relate to mostly refined white rice (without bran {the fibrous outer layer}) and germ (the nutritious core) having less nutrition than whole grain rice) [23].

In 2014, Mozaffarian et al. [24] attributed 1.65 million cardiovascular deaths worldwide to sodium consumption above 2.0 g per day. However, based on a prospective cohort study, O’Donnell et al. [25] reported an optimal average sodium intake range of 3-5 g/day, with cardiovascular events most prominently associated with higher sodium intake (>5 g/day) in those with hypertension. The joint working group of the World Heart Federation, the European Society of Hypertension, and the European Public Health Association in 2017 [26] concluded that the guidelines restricting sodium intake were far too restrictive.

In this GBD analysis, Japanese had the world’s highest mean sodium (gram/day) (sodium=6.01 g/day versus global average sodium {gram/day}=4.45 g/day, Table 5). Japanese also had a relatively high prevalence of smoking (smoking prevalence=26.8%) and the lowest mean NCDs in the world after Kuwait (mean Japanese NCDs=725.61 deaths/100000/year). Even a 5 g/day guideline may not be needed for people without medical indications for restrictions on sodium intake. The American Heart Association might note these GBD data in reconsidering sodium intake recommendations.

The worldwide negative correlation of LDL cholesterol with NCDs was also unexpected (r, -0.339; 95% CI, -0358 to -0.319; p<0.0001, Table 5). In the high animal food seven subset (NCDs of <1070.23 or animal food seven of >400 KC/d, n=1722 cohorts m/f, Table 15), the LDL cholesterol correlated weakly positively with NCDs (r, 0.137; 95% CI, 0.090-0.183; p<0.0001), and the multiple regression formula from this subset attributed 0.28 PAR% to LDL cholesterol. However, in Table 17, with the same subset of mostly high animal food male and female cohorts unpaired, LDL cholesterol was not significantly correlated with NCDs (r, 0.027; 95% CI, -0.021-0.074; p=0.2702).

Controversy about drug treatment of high LDL cholesterol has appeared in recent literature. In the British Medical Journal (BMJ), in 2016, Ravnskov et al. [27] published a systematic review of LDL cholesterol and mortality in the elderly that showed no association or an inverse association. A BMI evidence-based medicine article by DuBroff et al. [28] called into question using LDL cholesterol to justify drug treatment for cardiovascular disease. The GBD global data on LDL cholesterol inversely correlating with NCDs should weigh into the current controversy, and the American Heart Association might reconsider their guidelines for drug treatment of high LDLc. Reductions in meat, dairy, poultry, and added SFA should obviate the need for drugs except for genetically caused severely high low-density lipoprotein hypercholesterolemia.

The non-dietary risk factors in the multiple regression risk factor formula have plausible PAR%s given whether the subset analyzed had low animal food seven (Tables 10-13) or high animal food seven (Tables 15-17). As might be expected, vitamin A deficiency in children, severe underweight in children, ambient air pollution, and household air pollution were prominent in the low animal food seven/low sociodemographic index (mean SDI=0.411) cohorts. Smoking prevalence appeared only when cohorts were unpaired (Table 14), allowing the higher NCDs and higher smoking prevalence in males to have full influence.

In the high animal food seven/high sociodemographic index cohorts (mean SDI=0.750, Tables 15-17), the major non-dietary risk factors were stopping breast feeding before six months, smoking prevalence, lead, and body mass index.

The limitations of this study included using observational data, which can only show association and cannot establish causation between risk factors and NCDs. Also, this study focused on the relationship between diet and NCDs at the population level and did not provide individual-level analysis. Our study was subject to all the limitations discussed in previous GBD publications [29,30]. These included gaps, biases, and inconsistencies in data sources, as well as limitations in the methods of data processing and estimation. Having comprehensive data on dietary inputs is key to more accurate and reliable analyses. These GBD data on animal foods, plant foods, alcohol, sugary beverages, and fatty acids were not comprehensive and comprised only 1218.98 KC/d per person on average worldwide (Table 5). Subnational data on all risk factors were available in only four countries. Because the data formatting and statistical methodology were new, this was necessarily a post hoc analysis, and no pre-analysis protocol was possible. We and other researchers should repeat this GBD data analysis when the IHME releases the GBD2021 data and make them available to IHME volunteer collaborators.

## Conclusions

GBD data modeling supported many but not all of the PHD dietary recommendations. This evidence-based methodology of analyzing IHME GBD data may have advantages over systematic literature review studies in developing health policy strategies, clinical practice guidelines, and public health recommendations. First, using a form of artificial intelligence (a large dataset from 195 countries), this study provided comprehensive analyses of the relationship between dietary and non-dietary risk factors and NCDs in selected subsets. Second, it provided estimates of optimal ranges of food risk factors for minimizing NCDs, using a methodology that can apply to individual noncommunicable diseases (e.g., colon cancer, ischemic heart disease, or BMI). Third, the multiple regression analyses provided quantitative formulas for estimating the risk of NCDs based on various risk factors in selected subsets of the GBD data. This can be useful for identifying high-risk populations and targeting interventions. Last but not least, this study included data on 20 low-NCD countries with relatively low animal food intake (mean m/f animal food seven of <400 KC/d). This can be helpful for identifying dietary and lifestyle patterns that may be protective against NCDs or other health outcomes (e.g., BMI). It can also lead climate scientists to learn from countries that have limited greenhouse gas emissions from animal foods while achieving low NCDs.

## Appendices

Appendix 1: Methodology for deriving multiple regression risk factor formulas

Table 20 provides an overview of the steps in deriving the multiple regression risk factor formula

**Table 20:** Overview of the steps in the derivation of the multiple regression risk factor formula NCD, noncommunicable disease; KC/d, kilocalories/day; PAR%, population-attributable risk percent

Step	Name	Intent/purpose 1	Intent/purpose 2
1	Select the subsets to be used for deriving multiple regression formulas.	Evaluate a low animal food seven cohort subset.	Evaluate a high animal food seven cohort subset.
2	Integrate Statistical Analysis System (SAS) and Excel (Microsoft^®^ Corp., Redmond, WA) (spreadsheets) for the analysis.	Use SAS for formatting data and performing calculations.	Use Excel for entering and manipulating SAS results and returning manipulated results to SAS.
3	Standardize all risk factors and NCDs.	Puts risk factor impacts on NCDs on comparable scales.	Enhances the validity of using multiple regression analyses.
4	Create a combination variable of 20 dietary risk factors.	Minimize confounding by multicollinearities.	Include as many dietary variables as are available.
5	Create a combination variable of relevant non-dietary risk factors.	Minimize confounding by multicollinearities.	Exclude non-dietary variables that are confounded by multicollinearities.
6	Multiply each dietary risk factor by its mean KC/d in the subset of interest and the R^2^ of the correlation with NCDs.	Weight each dietary risk factor’s PAR% in the eventual multiple regression formula by the KC/d of that risk factor.	Weight each dietary risk factor’s PAR% in the eventual multiple regression formula by the strength (R^2^) of that risk factor’s correlation with NCDs.
7	Multiply each non-dietary risk factor with global scope by one (e.g., air pollution), and multiply each non-dietary risk factor affecting a fraction of the population by the fraction (0-1, e.g., smoking).	Weight each non-dietary risk factor’s PAR% in the eventual multiple regression formula by the portion of the population affected.	Weight each non-dietary risk factor’s PAR% in the eventual multiple regression formula by the strength (R^2^) of the correlation of that risk factor with NCDs.
8	Empirically develop methods to eliminate or attenuate confounding by multicollinearities.	Move dietary risk factors with implausible signs (e.g., fruit positively correlated with NCDs), and make them independent risk factors in the multiple regression analysis.	Remove non-dietary risk factors with implausible signs (e.g., physical activity positively correlated with NCDs).
9	Perform multiple regression analyses with the dietary and non-dietary risk factor combination variables and independent variables (e.g., sex and confounded dietary risk factors).	Derive the parameter estimates and the partial R^2^ of all the variables.	Multiply each dietary and non-dietary risk factor by its parameter estimate or its partial R^2^ based on empirical judgement.
10	From the above, derive a preliminary NCD risk factor formula and a final risk factor formula.	Copy the preliminary risk factor formula into Excel.	Algebraically equate the preliminary risk factor formula into the final risk factor formula.

Our multiple regression formula derivation method differed from standard modeling in several important ways. We did not seek to minimize the number of individual dietary and non-dietary risk factors included or to maximize the total variance (and population-attributable risk percents {PAR%s}) of each formula. Instead, we developed several strategies to combat the confounding of risk factors by risk factor to risk factor interactions and to enhance the plausibility of each risk factor’s PAR%.

GBD analysis database subsets were used to derive two risk factors versus NCD multiple regression formulas: (1) The first analysis included those cohorts with mean (m/f pairs) animal food seven of <400 KC/d out of the 500 pairs of the lowest NCD cohorts (1000 cohorts, representing about one billion people) and all other mean m/f cohorts with animal food intake less than the mean m/f animal food consumption of the lowest NCD country in the subset (e.g., Kenya). (2) The second included all 500 m/f pairs of cohorts with the lowest NCDs and all other cohort pairs with mean animal food seven consumption of ≥400 KC/d (i.e., all 500 pairs of cohorts in the lowest NCD subset and say 500-1500 pairs of other cohorts with mean m/f animal food seven of ≥400 KC/d).

Using Statistical Analysis System (SAS) and Excel (spreadsheets), the resulting multiple regression analysis-derived formulas with dietary and other risk factors (independent variables) for NCDs (dependent variable) came from these subsets. Since there was no published ecological epidemiologic methodology to derive PAR%s for each of >20 risk factors, we used the following empirically developed 10 steps in the multiple regression analyses:

(1) We standardized all dietary and non-dietary risk factors. This made the impacts of all dietary and non-dietary risk factors on the same scale measured in standard deviations (SDs) from the mean.

(2) We created a combination of dietary risk factor variable composed of adding together 20 dietary risk factors that each had the following adjustments: (a) multiply each risk factor times its mean kilocalories/day (KC/d), quantifying the proportion of the dietary impact of each dietary risk factor’s share of the PAR% related to NCDs attributable to KC/d exposure, and (b) multiply each risk factor times the R^2^ (coefficient of determination) of its univariate correlation with NCDs, quantifying the portion of the PAR% attributable to the strength of the R^2^ of the risk factor with NCDs.

(3) Should one or more dietary risk factor coefficient’s sign(s) be questionable and multicollinearities with other risk factors suspected, our empirically derived indications for removing the dietary risk factor(s) from the dietary combination variable and making it/them independent variable(s) in the multiple regression were the following: (a) Animal foods: all signs are determined according to their correlations (r signs) with NCDs. (b) Alcohol: if a negative sign, take out of the combination diet variable, and make it an independent variable in the multiple regression. (c) Sugary beverages: if a negative sign, take out of the combination diet variable, and make it an independent variable in the multiple regression. (d) Added TFA, potatoes, corn, and rice: determine the formula signs according to its/their univariate correlations (r sign{s}) with NCDs. (e) Healthy plant food seven individual risk factors (fruits, vegetables, nuts and seeds, whole grains, legumes, sweet potatoes, and added PUFA) with positive sign(s): take the risk factor(s) out of the combination diet variable, and make it/them independent variable(s) in the multiple regression.

(4) Once all dietary risk factors in Excel were adjusted by 2a to 2b and 3a to 3e, the dietary risk factor combination variable was copied from Excel to SAS for multiple regression analyses with NCDs.

(5) For (1) any metabolic risk factors and (2) other non-dietary risk factors, we separately performed steps 1, 2, and 3 with some modifications. Because of the close correlations of metabolic risk factors with diet, we separated these groups into two combination variables. Sex (male and female) was always included as an independent risk factor in the multiple regression. Included and excluded non-dietary risk factors had the following univariate correlation signs with NCDs: (a) For metabolic risk factors (body mass index {BMI}, fasting plasma glucose {FPG}, low-density lipoprotein cholesterol {LDLc}, and systolic blood pressure {SBP}), “+” signs increased NCD risk, and “-” signs resulted in exclusion due to confounding. (b) For physical activity, “-” reduced NCD risk, and “+” resulted in exclusion because physical activity was confounded by other variables. (c) For vitamin A deficiency in children of <5 years old/100000/year, “+” increased NCD risk, and “-” resulted in risk factor exclusion due to confounding. (d) For childhood severe underweight of >2 standard deviations (SDs) below the World Health Organization (WHO) mean, “+” increased NCD risk, and “-” resulted in risk factor exclusion due to confounding. (e) For stopped breast feeding before six months, “+” increased NCD risk, and “-” resulted in risk factor exclusion due to confounding. (f) For toxins (e.g., smoking, ambient air pollution, and lead exposure), “+” increased NCD risk, and “-” resulted in risk factor exclusion from the formula due to confounding. (g) For sex (male=1, and female=2), if females had fewer NCDs, then the sign is “-,” and if there were fewer male NCD deaths, then sign is “+.”

(6) For the metabolic and other non-dietary risk factors in the two combination variables, we set the risk factor exposures as follows: (a) If the risk factor applied to all people in the cohort (e.g., physical activity, metabolic risk factors, or air pollution), we set the mean exposure at one, and (b) if the risk factor applied only to a portion of the people in the cohort (e.g., smoking), we set the mean exposure at 0-1, the portion of the people exposed (e.g., smoking mean exposure=0.20, representing 20%), or for vitamin A deficiency per 100000 children, the exposure was mean incidence (e.g., 20000/100000=0.200).

7. As with the dietary risk factors’ adjustments for the strength of NCD correlations, we measured the strength(s) of the NCD correlation(s) of metabolic risk factors and the other non-dietary risk factors by the R^2^. If no metabolic risk factors were positively correlated with NCDs, then there was one non-dietary combination risk factor variable.

8. We then copied the one or two non-dietary risk factor combination variable(s) resulting with the dietary risk factor combination variable from SAS to Excel.

9. Next, we performed multiple regression analyses with NCDs (dependent variable) versus the dietary risk factor combination variable and the one or two non-dietary risk factor combination variable(s), all adjusted for the exposures of risk factors (e.g., KC/d) and strengths of NCDs-risk factors’ R^2^ correlations. Any omitted dietary variables that became independent variables (e.g., dietary risk factors, sugary beverages, or alcohol that were negatively correlated with NCDs) were also individually included in the multiple regression along with sex (male=1, and female=2). Multiple regressions with mean values of m/f pairs of risk factors did not include sex as a risk factor: (a) Taking the SAS multiple regression results back to the Excel spreadsheet, we multiplied each risk factor times its exposure (i.e., step 2a), times the strength of its R^2^ with NCDs (i.e., step 2b), and times the corresponding parameter estimates from the dietary and non-dietary combination risk factor variables. (b) If dietary risk factors were shifted from the combination variable per step 5 to become independent variables, they would be multiplied by the partial R^2^ instead of the parameter estimate to capture only the additional total formula R^2^ they contributed to the risk factor formula. An implausible dietary risk factor sign (e.g., “-” for sugary beverages or alcohol) might be reversed when the risk factor became an independent variable in the multiple regression. All independent risk factors would have as their coefficients their partial R-squared values. If implausible risk factor(s) signs were not reversed in the multiple regression, the partial R-squared coefficient(s) would be reversed in the plausible direction. (c) Step 9a-b created a single combination risk factor variable composed of all the dietary and non-dietary risk factors. We called this preliminary risk factor formula 1 and copied it into a data step in SAS.

10. With the single combination risk factor variable derived in step 9, we performed the following steps to equate the risk factor coefficients to their PAR%s: (a) In Excel, we totaled the risk factor coefficients of the single combination risk factor variable (“preliminary risk factor formula 1”). (b) We determined the correlation (r) of the preliminary risk factor formula 1 in SAS, copied it into Excel, and subsequently calculated the R^2^ of the risk factor formula. (c) We then divided the preliminary risk factor formula 1’s R^2^ (step 10b) by the sum of the absolute values of the risk factor coefficients (step 10a) to generate a multiplier. (d) We copied preliminary risk factor formula 1 onto an adjacent location in Excel in preparation to equate the risk factor coefficients to their PAR%s by using the multiplier. (e) We then multiplied each risk factor coefficient in step 10d with the multiplier. (f) We multiplied times 100 to derive the final risk factor formula with coefficients equated to final PAR%s. (g) Finally, we then took the final risk factor formula from step 10f to the PROC CORR function in SAS to confirm that it had the same r and R^2^ as preliminary risk factor formula 1.

Appendix 2: Methodology for deriving the optimal ranges of dietary risk factors (KC/d)

Table 21 provides a methodology synopsis.

**Table 21:** Synopsis of the methodology for deriving the GBD optimal food KC/d ranges GBD, Global Burden of Disease; KC/d, kilocalories/day; m/f, male/female

Step	Name	Intent/purpose 1	Intent/purpose 2
1	Select the subsets to be used for deriving the optimal ranges for dietary risk factors (KC/d).	Select a low animal food seven subset.	Select a high animal food seven subset.
2	Adjust the mean (m/f) KC/d values of risk factors in the low and high animal food data subsets as determined empirically.	If r was -, add R^2^ to the mean (m/f) KC/d risk factor value.	If r was +, subtract R^2^ from the mean (m/f) KC/d risk factor value.
3	Switch low and high animal food subset risk factor(s) KC/d(s) adjusted values as appropriate.	Find risk factor KC/d values from the low animal foods that are higher than the value in the high animal food subset.	Switch the values in the low animal food subset as needed to keep all risk factor values in KC/d lower than in the high animal food subset.
4	For each dietary risk factor, derive the mean m/f KC/d.	For each dietary risk factor, find the means of the low and high animal food subset values.	

From the two GBD subsets used in deriving the multiple regression risk factor formulas (Appendix 1), we derived optimal range estimates for 22 dietary risk factors (including animal food seven and healthy plant seven) with the following steps:

1. From the GBD subsets defined above for the two multiple regression formulas with pairs of cohorts (male and female mean KC/d and mean m/f NCDs), we kept the mean KC/d values and the risk factor to NCD R^2^ values (coefficients of determination).

2. We then adjusted the mean (m/f) KC/d values of risk factors in the low and high animal food data subsets by the following: (a) Using 1±R^2^ depending on the sign of the r (+ if r was - and - if r was +), for each dietary risk factor, we added or subtracted the R^2^ of each dietary risk factor versus NCDs (i.e., 1±R^2^). (b) We calculated each dietary risk factor’s adjusted mean KC/d value by multiplying it times 1±its R^2^s in univariate correlations with NCDs (i.e., dietary risk factor mean KC/d*(1±its R^2^). For example, if red meat = 20 KC/d and the red meat R^2^ with NCDs=-0.400, the adjusted red meat mean=20*(1+0.400)=28 KC/d, constituting the adjusted lower boundary of the optimum range for red meat. (c) Repeat step 2a-b for the dietary risk factors in the high animal food subset. For example, if mean red meat=100 KC/d and red meat R^2^ with NCDs=+0.400, the adjusted red meat mean=100*(1-0.400)=60 KC/d, constituting the upper boundary of the optimum range for red meat. (d) With the adjusted mean values for dietary risk factors from the low and high animal food subsets juxtaposed, any dietary risk factors from the low animal food subset that was higher than the corresponding risk factor in the high animal food subset would result in switching the values. This gave the final upper and lower boundary values of the optimal range for the risk factors.
